# A disulfidptosis-related lncRNA signature for analyzing tumor microenvironment and clinical prognosis in hepatocellular carcinoma

**DOI:** 10.3389/fimmu.2024.1412277

**Published:** 2024-10-07

**Authors:** Haishui Zheng, Jigan Cheng, Ziyun Zhuang, Duguang Li, Jing Yang, Fan Yuan, Xiaoxiao Fan, Xiaolong Liu

**Affiliations:** ^1^ Department of General Surgery, Sir Run Run Shaw Hospital, School of Medicine, Zhejiang University, Hangzhou, China; ^2^ Shantou University Medical College, Shantou, China; ^3^ Department of Breast Cancer, Cancer Center, Guangdong Provincial People's Hospital.Guangdong Academy of Medical Sciences, Guangzhou, China

**Keywords:** hepatocellular carcinoma, disulfidptosis, lncRNA, tumor microenvironment, SLC7A11

## Abstract

**Introduction:**

Disulfidptosis is a recently identified form of non-apoptotic programmed cell death which distinguishes itself from classical cell death pathways. However, the prognostic implications of disulfidptosis-related long non-coding RNAs (DRLs) and their underlying mechanisms in hepatocellular carcinoma (HCC) remain largely unexplored.

**Methods:**

In this study, we leveraged RNA-sequencing data and clinical information of HCC patients from the TCGA database. Through expression correlation and prognostic correlation analyses, we identified a set of top-performing long non-coding RNAs. Subsequently, a 5-DRLs predictive signature was established by conducting a Lasso regression analysis.

**Results:**

This signature effectively stratified patients into high- and low-risk groups, revealing notable differences in survival outcomes. Further validation through univariate and multivariate Cox regression analyses confirmed that the risk score derived from our signature independently predicted the prognosis of HCC patients. Moreover, we observed significant disparities in immune cell infiltration and tumor mutation burden (TMB) between the two risk groups, shedding light on the potential connection between immune-related mechanisms and disulfidptosis. Notably, the signature also exhibited predictive value in the context of chemotherapeutic drug sensitivity and immunotherapy efficacy for HCC patients. Finally, we performed experimental validation at both cellular and patient levels and successfully induced a disulfidptosis phenotype in HCC cells.

**Discussion:**

In general, this multifaceted approach provides a comprehensive overview of DRLs profiles in HCC, culminating in the establishment of a novel risk signature that holds promise for predicting prognosis and therapy outcomes of HCC patients.

## 1 Introduction

Hepatocellular carcinoma (HCC) is a multifaceted, globally impactful disease (1). Owing to its inconspicuous clinical symptoms in the early stages and the presence of numerous risk factors, it poses a significant clinical challenge. Primary liver cancer is the sixth most common cancer in the world and the third leading cause of cancer-related deaths in 2022, according to global cancer statistics published in 2024 (2). HCC accounts 80-90% of primary liver cancer cases and is the most common type (3). In recent years, enormous strides in targeted therapies and immunotherapies have been developed, offering renewed hope for better patient outcomes (4). However, patients with HCC continue to experience high rates of recurrence, metastasis, and drug resistance, contributing to an unfavorable prognosis (5). This underlines the necessity for early detection and a multidisciplinary approach for managing this formidable malignancy. Therefore, it is imperative to explore the intricate molecular mechanisms underlying HCC. The development of innovative therapies is indispensable in the ongoing battle against HCC.

Cell death is crucial for the development and homeostasis of multicellular organisms, and its dysregulation can lead to various diseases including cancer (6). A comprehensive understanding of programmed cell death modes could potentially pave the way for the targeted elimination of cancer cells, thereby improving cancer treatment outcomes (7). For example, the apoptotic signaling pathway has been the focal point of tumor chemotherapy in the past few decades. Some chemotherapeutic drugs, such as Paclitaxel and Vinca Alkaloids, can induce apoptosis of tumor cells by targeting microtubules and oxidative phosphorylation, thus achieving a therapeutic effect on tumors (8, 9). However, these therapies are associated with a high rate of drug resistance, posing significant challenges. Therefore, it is necessary to expand our understanding of regulated cell death modes beyond apoptosis to facilitate the discovery of potential therapeutic targets. In recent years, research has increasingly shown that many forms of non-apoptotic cell death are also executed in a regulated manner, which are collectively referred to as “regulated non-apoptotic cell death modes” (10). These newly named cell death modes include necroptosis, oxytosis, pyroptosis, parthanatos, NETosis, ferroptosis and cuproptosis (11–16). Recently, Gan et al. proposed a novel form of cell death known as disulfidptosis, which opens a promising avenue for cancer treatment. Disulfidptosis is triggered by the significant accumulation of disulfide molecules within cancer cells in the absence of glucose, particularly in those with elevated SLC7A11 expression. This phenomenon results in abnormal disulfide bonding between actin cytoskeletal proteins, disrupting their organization, and ultimately leading to the collapse of the actin protein network and cell death. Gan et al. treated cancer cells with a glucose transporter (GLUT) inhibitor and observed that the outcome was similar to that under glucose-deprived conditions (17). This inventive discovery has immense potential for developing targeted therapies for cancer treatment. It is crucial to acknowledge that the comprehension of distinct forms of cell death, including apoptosis, necrosis, ferroptosis, and the newly discovered disulfidptosis, remains an evolving frontier in the realm of cell biology and cancer research. Further investigations are warranted to comprehensively elucidate disulfidptosis, its relevance in the context of cancer, and its potential as a target for therapeutic interventions.

LncRNAs are a class of non-coding RNA molecules with a length exceeding 200 nucleotides. This type of RNA lacks an open reading frame (ORF) and does not encode proteins, leading to the belief that it exists solely as a transcriptional byproduct. However, extensive research has demonstrated that lncRNAs participate in various biological processes including DNA methylation, histone modification, post-transcriptional regulation of RNA, and protein translation (18). Additionally, lncRNAs play pivotal roles in processes related to immunology, neurobiology, inflammatory responses, and cancer (19). Furthermore, lncRNAs are critical regulators of cellular proliferation and programmed cell death. Sun et al. discovered that lncRNA-ATB regulates the formation of tumor metastasis foci by modulating the stability of IL-11 mRNA and STAT3 phosphorylation (20). Concerning regulated cell death, lncRNA-HEPFAL was found to promote ferroptosis by reducing SLC7A11 expression and increasing levels of lipid reactive oxygen species (ROS) and iron ions (21). The association between lncRNAs and HCC has been extensively explored, notably in the regulation of cell death processes in HCC. For example, Chen et al. revealed that lncRNA DUXAP8 decreased the sensitivity of HCC to sorafenib-induced ferroptosis by interacting with SLC7A11 (22). In the era of precision medicine, identification of precise lncRNAs that regulate disulfidptosis in HCC, along with a thorough elucidation of their mechanisms, could offer innovative insights and perspectives for the treatment of HCC.

In this study, we collected HCC data from The Cancer Genome Atlas (TCGA) database to elucidate the prognostic and biological functions of disulfidptosis-related long non-coding RNAs (DRLs) through various bioinformatic analyses. Our 5-DRLs signature exhibited excellent performance in predicting patient survival and remarkable superiority over the other clinically independent variables. Additionally, we established a potential relationship between the risk signature and tumor microenvironment (TME), as well as tumor mutation burden (TMB), through immune infiltration analysis and TMB analysis. Furthermore, KEGG and GO analyses were performed between the high- and low-risk groups to identify the potential molecular pathways. Overall, our findings shed light on the understanding of molecular mechanisms related to disulfidptosis in HCC and could help to develop individualized therapies for patients with HCC.

## 2 Materials and methods

### 2.1 Data acquisition

A total of 374 HCC samples and 50 adjacent normal hepatic sample fragments per kilobase of transcript per million mapped reads (FPKM)-standardized RNA-seq data were downloaded from The Cancer Genome Atlas(TCGA) (https://portal.gdc.cancer.gov/projects/TCGA-LIHC). Ensembl IDs were processed and converted to official gene symbols encompassing various elements, such as lncRNAs, protein-coding genes, and miRNAs. Additional information, including clinical data, was also acquired from patients with HCC in the TCGA database. Samples lacking survival information and those with an overall survival (OS) of less than 30 days were excluded from subsequent analysis. Ten Disulfidptosis-related Genes (DRGs) were obtained in a recent study (**Table 1**) (17).

**Table 1:** Disulfidptosis-related genes.

Official Symbol	Official Full Name
GYS1	Glycogen Synthase 1
NDUFS1	NADH Dehydrogenase Fe-S Protein 1
OXSM	3-Oxoacyl-ACP Synthase, Mitochondrial
LRPPRC	Leucine Rich Pentatricopeptide Repeat Containing
NDUFA11	NADH: Ubiquinone Oxidoreductase Subunit A11
NUBPL	NUBP Iron-Sulfur Cluster Assembly Factor, Mitochondrial
NCKAP1	NCK Associated Protein 1
RPN1	Ribophorin I
SLC3A2	Solute Carrier Family 3 Member 2
SLC7A11	Solute Carrier Family 7 Member 11

### 2.2 Identification of disulfidptosis co-expressed lncRNAs

Pearson’s correlation analysis was chosen as a widely accepted method to explore the correlation between coding genes and lncRNAs. Using a cutoff criterion of R > 0.3 and *P* < 0.001, Pearson correlation analysis was applied to identify lncRNAs that were co-expressed with the 10 DRGs from the RNA-seq data of TCGA HCC samples.

### 2.3 Identification of differentially expressed lncRNAs

Differentially expressed lncRNAs between HCC and normal patients from TCGA were identified using the R package “Limma.” The significance criterion for identifying DEGs was set as |log_2_ (fold-change) | > 1 and *p* < 0.001.

### 2.4 Univariate cox analysis for prognostic lncRNAs

By using the ‘survival’ R package and defining *p* < 0.05 as screening criteria, the intersecting lncRNAs of DCLs and DELs were subsequent to univariate cox analysis for obtaining prognostic DRLs in HCC patients.

### 2.5 Construction and validation of the disulfidptosis-related prognostic signature

To construct a disulfidptosis-related prognostic signature, least absolute shrinkage and selection operator (LASSO) Cox regression analysis was used to select the most appropriate lncRNAs and estimate and weight the regression coefficients of the optimal DRLs (23). Initially, nine prognostic lncRNAs were screened based on the optimal penalty parameter λ determined by tenfold cross-validation following the minimum criteria. Afterwards, a multivariate Cox regression analysis was conducted to establish a five-lncRNA predictive model. The Risk score of each HCC patient was calculated using the following formula: Risk score = (Coef.DRL1 × DRL1 exp.) + (Coef.DRL2 × DRL2 exp.) + (…) + (Coef. DRLn × DRLn exp.). Patients were categorized into low- and high-risk groups based on the median risk score. The performance and prognostic ability of the predictive signature were evaluated using time-dependent receiver operating characteristic (ROC) analyses and Kaplan-Meier log-rank tests. These analyses were conducted with the R packages “timeROC” and “survival,” respectively (24). Furthermore, in combination with the DRL prognostic signature, the clinical characteristics of patients with HCC from TCGA were analyzed using univariate and multivariate Cox regression analyses.

### 2.6 Establishment of a nomogram

Package “rms” was utilized to create a nomogram, offering valuable clinical prognostic insights for HCC patients, including their risk scores and various clinicopathological attributes, particularly about 1-, 3-, and 5-year OS (25). Subsequently, we conducted calibration curve analysis to validate the clinical accuracy of the nomogram.

### 2.7 Relationship of DRL risk signature with tumor microenvironment in HCC

The immune and stromal scores of each HCC patient were calculated using the ESTIMATE algorithm (26). Next, the levels of 22 immune cell subtypes of each patient were computed using the CIBERSORT algorithm (27). The differentially expressed immune checkpoint genes between high- and low- risk groups were identified using R package “Limma.” Immunophenoscore (IPS) was obtained from the TCIA database (https://tcia.at/home) to predict the relative immune response (28).

### 2.8 Tumor mutation burden analysis

To delineate the mutational profiles of HCC patients within two distinct risk groups, the Mutation Annotation Format (MAF) was generated using the “maftools” package (29). This MAF served to characterize the mutational landscape of patients with HCC from different DRL risk groups.

### 2.9 Drug sensitivity analysis

The semi-inhibitory concentration (IC50) values for commonly used chemotherapy agents in HCC patients were calculated using the “pRRophetic” package to predict the clinical performance of chemotherapy agents in different DRL risk groups for HCC patients (30).

### 2.10 Gene set enrichment analysis, Kyoto encyclopedia of genes and genomes and gene ontology analysis

To identify the potential molecular pathways between the high- and low-risk groups, KEGG, GO, and GSEA were performed. Firstly, R package “Limma” was performed to classified differential expressed genes between high- and low-risk groups (|log_2_ FC| > 1, *p* < 0.05). Significant genes were inserted into the Database for Annotation, Visualization, and Integrated Discovery (DAVID, https://david.ncifcrf.gov/) to enrich closely related metabolic pathways. GSEA was performed using GSEA software with c5.all.v7.4 symbols.gmt as a template. The criteria for statistical significance were nominal *p* < 0.05 and FDR< 0.25.

### 2.11 Cell culture and human samples

The normal human liver cell line MIHA and the human hepatocellular carcinoma cell lines HA22T, HCCLM3, HepG2, and JHH-7 were purchased from the Cell Bank of the Chinese Academy of Sciences. All cell lines were cultured in DMEM medium (Gibco, USA) containing 10% fetal bovine serum (FBS, Gibco) at 37°C in humidified air with 5% CO_2_. A total of 16 HCC samples and adjacent normal tissues were collected from patients with HCC who underwent surgical resection at the Sir Run Run Shaw Hospital (SRRSH), in accordance with the principles of the Declaration of Helsinki. Written informed consent was obtained from all the patients. All human samples were obtained after obtaining informed consent as approved by the Institutional Review Board of SRRSH, School of Medicine, Zhejiang University, Hangzhou, China (ethical code: 20210729-282).

### 2.12 RNA extraction and quantitative real-time PCR

RNA extraction was performed using an RNA-Quick Purification Kit (AG21023, Accurate Biology). Reverse transcription was conducted according to the protocol of the Eco M-MLV RT Premix Kit (AG11706, Accurate Biology). RT-qPCR was conducted on a QuantStudio 1 (Applied Biosystems, Thermo Fisher Scientific, USA) using the SYBR Green Premix Pro Tag HS qPCR kit (AG11701, Accurate Biology). Target gene expression was normalized to the endogenous control gene glyceraldehyde 3-phosphate dehydrogenase (GAPDH). The primers used in this study was listed in **Table 2**.

**Table 2:** Primers for RT-qPCR in this study.

Primer name	Sequence (5’-3’)
TMCC1-AS1-F	GGTAGGGTAGCAGGTCAGCATATC
TMCC1-AS1-R	TTGTCACAGGCCAGACTACCAG
FOXD2-AS1-F	TATGTGGTAGGGGACTCGCT
FOXD2-AS1-R	GGTTTCAAGTGGCGCTGTTT
LINC01063-F	CCTGAGCCTGGAAGGTGATT
LINC01063-R	TGACTGAGGTTCGCTGTGAC
SLC25A30-AS1-F	CAAGTGCCCCTCAGGATCTTC
SLC25A30-AS1-R	AATTTCTCTTCCACCTCCCAGTC
AC009283.1-F	GCATCTGAGCAGCTGTGCAGCA
AC009283.1-R	CCTCCTCATCATCCTCCTGTGGGT
GAPDH-F	CTCTGCTCCTCCTGTTCGAC
GAPDH-R	ACCAAATCCGTTGACTCCGA
SLC7A11-F	TCTCCAAAGGAGGTTACCTGC
SLC7A11-R	AGACTCCCCTCAGTAAAGTGAC

### 2.13 Cell counting kit-8 assay

HepG2 and JHH-7 cell viability was assessed using the CCK-8 reagent (Meilunbio, China), following the manufacturer’s instructions. Cells were seeded in 96-well plates at a density of 3000 cells/well in 100 μL of medium. Subsequently, CCK8 solution (10 μL) was added to each well at 3, 6, 12, 24, 36, and 48 hours after treatment with glucose-free DMEM. The cells were then further incubated at 37°C for 2 h. The absorbance of each well was measured at 450 nm wavelength using a spectrophotometer.

### 2.14 Confocal microscopic imaging of F-actin staining

HepG2 and JHH-7 cells were seeded in 24-well plates at a density of 20000 cells per well and treated with DMEM Medium without glucose for 24 h. For actin filament staining, cells were fixed for 30 min at room temperature with 4% paraformaldehyde and then permeabilized for 10 min with permeabilization buffer (0.1% Triton X-100 in PBS). Subsequently, the cells were incubated in darkness at room temperature for 1-2 hours with TRITC Phalloidin (Solarbio, CA1610). Afterward, the cells were then washed twice and mounted with antifade mounting medium containing DAPI (Beyotime, P0131). Finally, all fluorescence images were captured using a confocal microscope (LSM 880, Zeiss).

### 2.15 Drugs and reagents

Z-VAD-FMK (ZVF, S7023), ferrostatin-1 (Fer-1, S7243), necrostatin-1 (Nec-1, S8037), and N-acetyl cysteine (NAC, S5804) were purchased from Selleck. Tetrathiomolybdate (TTM, 323446) was purchased from Sigma. Tris (2-carboxyethyl) phosphine (TCEP, T2556) was purchased from Thermo Fisher. The concentration of ZVF, Fer-1, Nec-1, NAC, TTM and TCEP were 30μM, 10μM, 20μM, 1mM, 20μM, 1mM.

### 2.16 Western blotting

Proteins from cells were extracted using radioimmunoprecipitation assay (RIPA) buffer (Fude Biotech, China) containing protease inhibitors. Subsequently, protein concentrations were determined using a Bicinchoninic Acid (BCA) Protein Assay Kit (Meilunbio, China). A total of 20 μg of protein was subjected to sodium dodecyl sulfate–polyacrylamide gel electrophoresis and transferred to a 0.22 or 0.45 µm polyvinylidene difluoride (PVDF) membrane. PVDF membranes were then blocked in 5% skim milk for 2 h. Subsequently, samples were incubated with specific primary antibodies at 4°C overnight. The primary antibodies were as follows: SLC7A11 (82115-2-RR, Proteintech, Wuhan, China), GAPDH (AC002, Abclonal, Wuhan, China). Following this, membranes were incubated with the appropriate secondary antibodies for 2 h at room temperature. Finally, the protein bands were visualized with enhanced chemiluminescence (ECL) Western blotting substrate (Fude Biotech, China).

### 2.17 RNA interference

The small interference RNAs (siRNAs) was designed and synthesized in GenePharma(China), which could effectively knock down lncRNAs effectively. Cells were transfected with 100 nM of smart silencer for each well using the Lipofectamine™ 3000 transfection reagent (L3000015; Thermo Fisher, USA). After 48 hours of transfection, cells were collected and processed for RT-qPCR and other experiments. The sequences of the lncRNA siRNA were listed in **Table 3**.

**Table 3:** Sequences of siRNAs for related lncRNAs.

LncRNA name	sense(5’-3’)	antisense(5’-3’)
FOXD2-AS1-Homo-1	GAGGGACAGCCAAGAAUACTT	GUAUUCUUGGCUGUCCCUCTT
FOXD2-AS1-Homo-2	AGUCCCAGACAGGGUAACUTT	AGUUACCCUGUCUGGGACUTT
FOXD2-AS1-Homo-3	GUCAGGAACUAAAGGACUGTT	CAGUCCUUUAGUUCCUGACTT
LINC01063-Homo-1	AUCAAGCGGUGGCAGUUCATT	UGAACUGCCACCGCUUGAUTT
LINC01063-Homo-2	GGAAGGUGAUUGGCUAGAGTT	CUCUAGCCAAUCACCUUCCTT
LINC01063-Homo-3	UGCGAGCAUCAUGUUGCCUTT	AGGCAACAUGAUGCUCGCATT

### 2.18 Statistical analysis

All statistical analyses were conducted using R software (Version 4.1.2). Wilcox test was used to compare lncRNA expression levels between HCC and para-noncancerous tissues sourced from TCGA. Differences in the proportions of clinical features were assessed using the chi-square test. A paired t-test was used to compare data between HCC and adjacent normal tissues obtained in-house. Variances among multiple groups were analyzed using one-way ANOVA. Statistical significance was defined as a *p*-value < 0.05.

## 3 Results

### 3.1 Identification of disulfidptosis-related differentially expressed and prognostic lncRNAs in HCC

**Figure 1:**
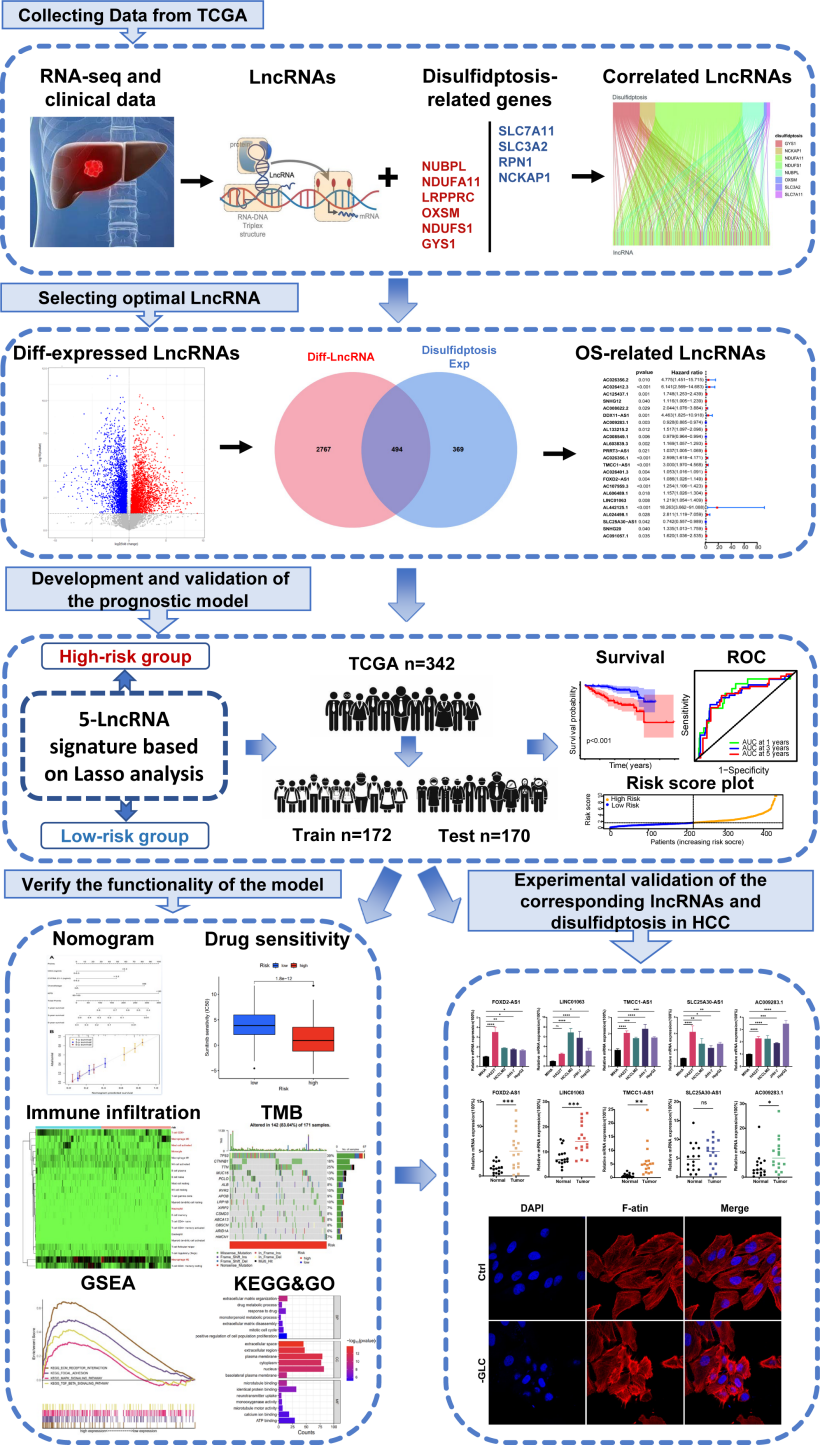
The flow chart of this study.

**Figure 2:**
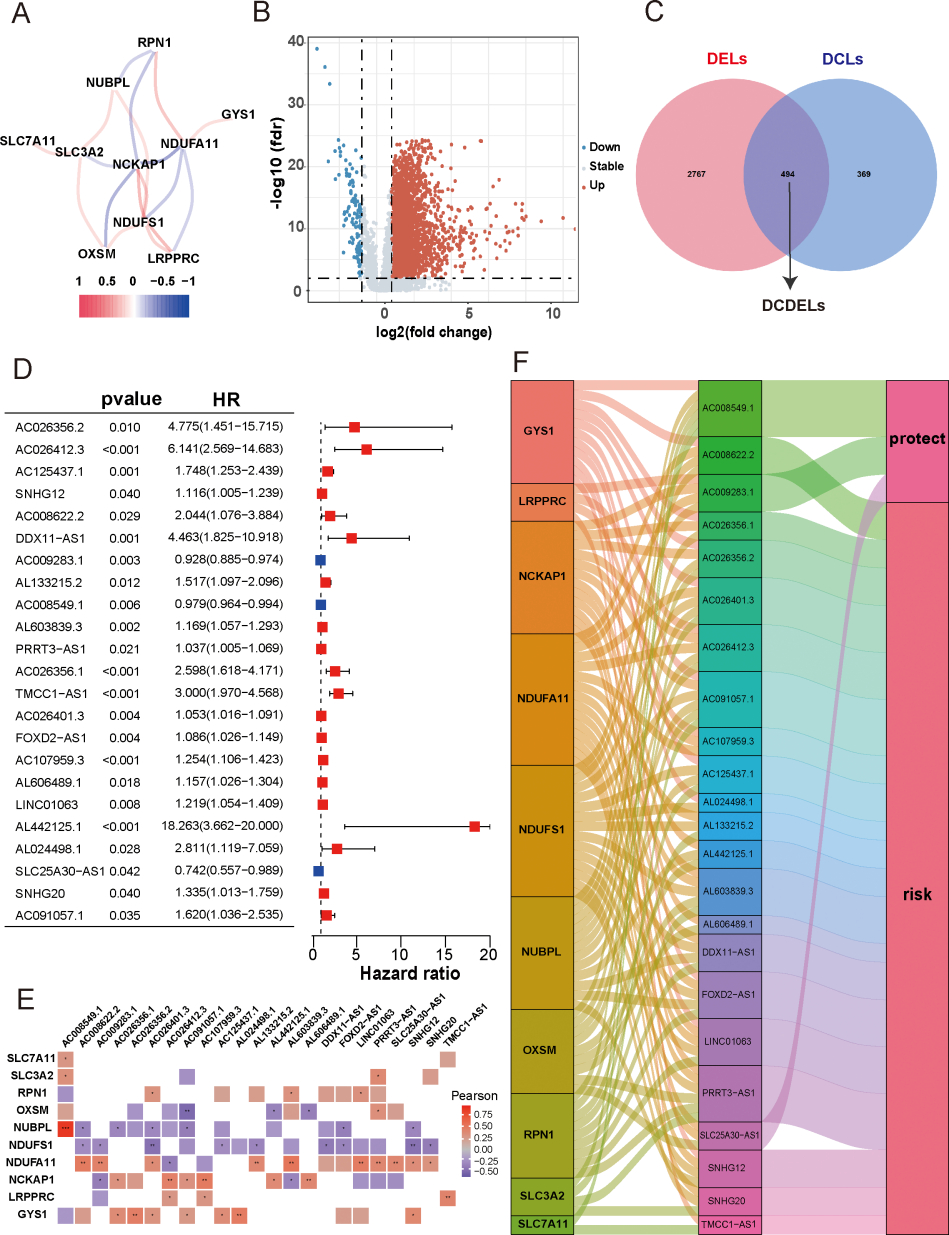
Identification of disulfidptosis-related differentially expressed and prognostic lncRNAs in HCC. **(A)** Co-expression network of 10 DRGs. (Red and blue colors represent a positive correlation and a negative correlation, respectively) **(B)** Volcano plot showed the differentially expressed lncRNA between the HCC tissues and adjacent normal tissues. (red: upregulated, blue: downregulated, grey: no significant) **(C)** Venn diagram displayed the lncRNAs shared by DCLs and DELs. **(D)** Forest plots presented the results of the univariate cox regression analysis of the 23 prognostic DCDELs. **(E)** Correlation of 23 prognostic DCDELs with 10 DRGs in TCGA-HCC Cohort. The color of each unit showed an indication of the degree of correlation. (Red implied a positive relationship, while blue indicated the opposite.) **(F)** The Sankey diagram demonstrated the roles of DCDELs and DRGs in HCC based on of Pearson’s R>0.3and *p*<0.001. HCC, hepatocellular carcinoma; DRGs, disulfidptosis-related genes; lncRNAs, long noncoding RNAs; DRLs, disulfidptosis-related long non-coding RNAs; DCLs, disulfidptosis co-expressed lncRNAs; DELs, differentially expressed lncRNAs; *p < 0.05, **p < 0.01, and ***p < 0.001.

Initially, we retrieved data from 374 patients diagnosed with HCC from TCGA database, consisting of transcriptomes and clinical information. Subsequently, we identified ten DRGs, as previously reported (**Table 1**) (17). The flowchart was presented in **Figure 1**. **Figure 2A** illustrated the correlation network diagram and provided insights into the interactions among these 10 DRGs in patients with HCC. To assess clinical relevance, we conducted a comparative analysis of gene expression between HCC tissues and adjacent normal tissues. A total of 3261 Differentially Expressed lncRNAs (DELs) were identified based on the criteria of |log_2_FC|>1, *p*<0.001. Detailed information on these DEGs were provided in **Supplementary Table 1**, and the volcano plot in **Figure 2B** depicted the variation in lncRNA expression levels between HCC and adjacent normal tissues. To investigate the relationship between DRGs and lncRNAs, we performed Pearson correlation analysis with a threshold of R>0.3 and *p*<0.001, leading to the identification of 863 DCLs, as shown in **Supplementary Figure 1**. The correlation between DRGs and lncRNAs were shown in **Supplementary Table 2**. We further integrated the DCLs and DELs, resulting in a set of 494 lncRNAs that were both differentially expressed and correlated, which named “DCDELs” (**Figure 2C**). Subsequently, we conducted a univariate Cox regression analysis to evaluate the prognostic ability of these DCDELs based on overall survival (OS) data from the TCGA clinical database. This analysis identified 23 prognostic DCDELs (**Figure 2D**, **Supplementary Table 3**). The correlation and differential expression between these prognostic DCDELs and DRGs were illustrated in **Figure 2E**, red indicates a positive correlation, while blue indicates a negative correlation. Furthermore, we generated a Sankey diagram (**Figure 2F**) to visually represent the roles of DCDELs and DRGs in HCC, providing a clear depiction of their correlation and prognostic significance in the context of patients with HCC.

### 3.2 Construction and validation of prognostic DRLs signature in HCC

**Figure 3:**
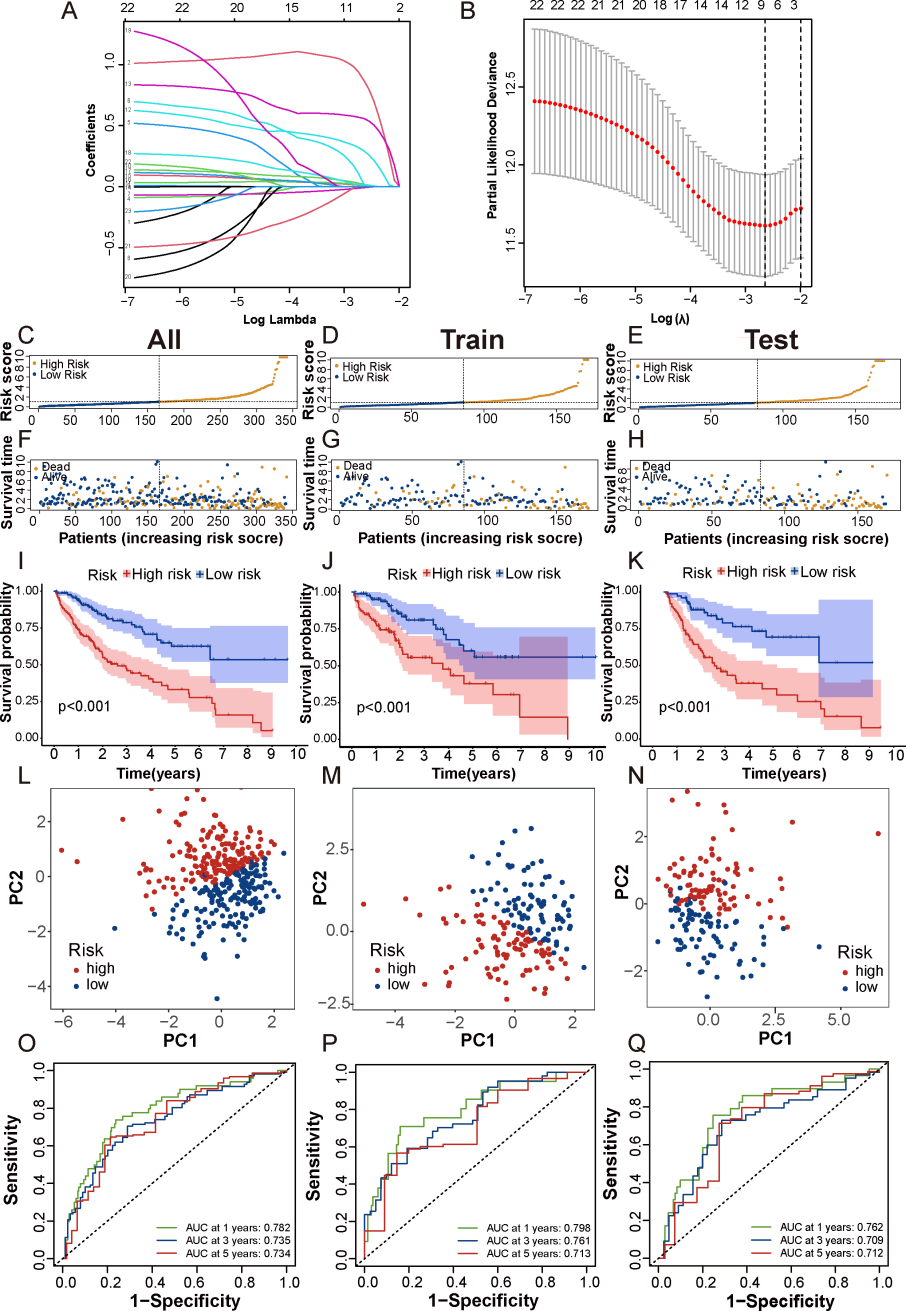
Construction and validation of prognostic DRLs signature in HCC. **(A, B)** Cvfit and lambda curves showed LASSO regression, constructed by the 10-fold cross-validation. **(C-E)** The distribution and median risk scores in the overall, train and test cohorts. **(F-H)** The distribution of overall survival status, survival time, and risk score in each cohort. **(I-K)** The Kaplan-Meier curves depicted the survival status and survival time of the overall, train and test cohorts. **(L-N)** PCA analysis showed a significant distinction in each cohort. **(O-Q)** AUC of the time-dependent ROC curves illustrated the ability of the signature consisting of DRLs to predict the 1-, 3- and 5-year OS in each cohort. lncRNAs, long noncoding RNAs; ROC, receiver operating characteristic; AUC, area under the curve; OS, overall survival.

First, we randomly divided 342 patients into training and test cohorts at a 1:1 ratio. Next, we conducted LASSO regression and multivariate Cox regression analyses to construct a prognostic signature based on the expression profiles of the previously identified 23 prognostic DCDELs (**Figure 3A**). **Figure 3B** illustrated the lambda curves obtained from LASSO regression analysis. LASSO regression selected nine lncRNAs based on the optimal penalty parameter λ, and multivariate Cox regression analysis further refined these to five lncRNAs, which were ultimately used to build the disulfidptosis-related prognostic signature. Ultimately, we identified five prognostic DRLs using the optimal penalty parameter λ determined through tenfold cross-validation following the minimum criteria. The risk score for each HCC patient was calculated using the following formula: Risk score = (1.583×TMCC1-AS1 expression) + (0.515×FOXD2-AS1 expression) + (0.577×LINC01063 expression) + (-0.698×AC009283.1 expression) + (-0.890×SLC25A30-AS1 expression) (detailed in **Supplementary Table 4**).

Based on the median risk score, patients were categorized into low- and high-risk groups (**Figures 3C-E**). To evaluate the feasibility and universality of the prognostic signature, we validated it in the train, test, and all cohorts. All cohorts exhibited a similar distribution in that the mortality rate increased in the high-risk score group, whereas the mortality rate decreased in the low-risk score group (**Figures 3F-H**). Furthermore, we compared the OS between the high-risk and low-risk groups using the Kaplan-Meier method, and the results revealed that the high-risk group had a significantly shorter OS than the low-risk group (*p* < 0.001) (**Figures 3I-K**). Principal Component Analysis (PCA) effectively discriminated the two risk subgroups in the train cohort, test cohort, and all cohorts (**Figures 3L-N**). The signature showed good performance in predicting survival in all cohorts (1, 3, and 5 years: AUC, 0.782, 0.735, and 0.734), in the train cohort (1, 3, and 5 years: AUC, 0.798, 0.761, and 0.713), and in the test cohort (1, 3, and 5 years: AUC, 0.762, 0.709, and 0.712) (**Figures 3O-Q**). Taken together, these findings demonstrated that this DRLs signature could serve as a reliable independent predictive tool for patients with HCC.

Additionally, when we compared progression-free survival (PFS) between the high- and low-risk groups, we observed that the PFS of the high-risk group was significantly lower than that of the low-risk group (**Supplementary Figures 2A, C, E**). Moreover, t-distributed Stochastic Neighbor Embedding (t-SNE) analysis revealed significant differences in distributions between the high- and low-risk groups in the overall dataset, test cohort, and train cohort (**Supplementary Figures 2B, D, F**).

### 3.3 Correlation between DRLs signature and clinicopathological features in HCC patients

**Figure 4:**
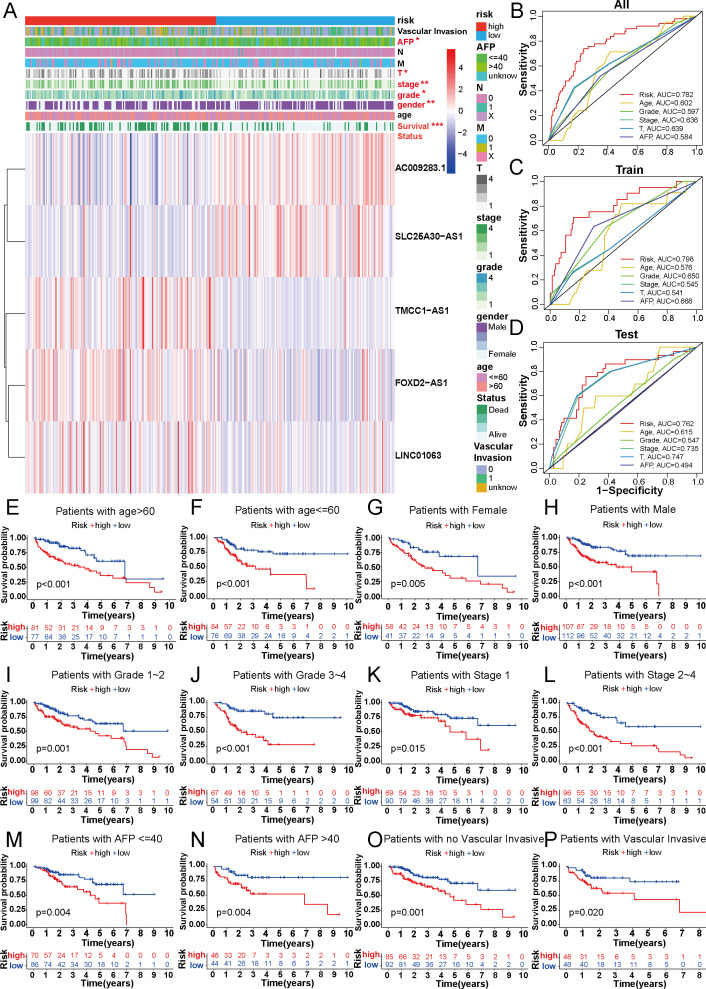
Correlation between DRLs signature and clinicopathological features in HCC patients. **(A)** The heatmap showed the distribution of ten specific clinicopathological characteristics and the corresponding risk score for the individual patient based on the predictive signature. Clinicopathological features highlighted in red indicated significant distinction in distribution between the high- and low-risk groups. **(B-D)** The ROC curves were performed to compare the prognostic accuracy of the signature and other prognostic parameters in overall, train and test cohorts. **(E-P)** Kaplan-Meier survival curves based on age, gender, grade classification, TNM stage, AFP and vascular invasive for high-risk and low-risk patient groups. *p < 0.05, **p < 0.01 and ***p < 0.001.

To explore the association between the 5-DRLs signature and disulfidptosis, we compared the expression levels of 10 DRGs between the low-risk and high-risk groups. The results indicated that the majority of DRGs expression exhibited distinct differences with significant p-values (**Supplementary Figure 3**). We further analyzed the connections in clinicopathological parameters between the two risk groups (**Figure 4A**). Significant variations were analyzed in factors such as Survival Status (*p*<0.001), gender (*p*<0.01), grade (*p*<0.05), T stage (*p*<0.05), stage (*p*<0.01) and AFP level (*p*<0.05) between the low- and high-risk groups and the differences were shown in **Supplementary Figure 4**. In addition, the 5-DRLs exhibited different distributions. AC009283.1 and SLC25A30-AS1 had higher expression levels in the low-risk group, while FOXD2-AS1, LINC01063, and TMCC1-AS1 showed the opposite trend. To further validate the performance of the 5-DRLs prognostic signature, we constructed ROC curves to demonstrate its superiority in terms of predictive accuracy compared to other clinicopathological parameters (**Figures 4B-D**). The results revealed that our risk signature exhibited excellent predictive performance, with AUC values of 0.782, 0.798, and 0.762 for the total, train, and test groups, respectively, which were significantly higher than those of other clinical univariate variables.

In addition, patients with HCC were categorized into different groups based on their age, gender, AFP level, tumor grade, TNM stage, and vascular invasion to verify whether our predictive model could be an effective supplement to the current staging system. For each group, the overall survival of the high-risk patients was remarkably lower than that of the low-risk group (**Figures 4E–P**). Model validation in different clinical subgroups indicated that the performance and predictive capability of the prognostic signature remained stable and effective under specific clinical conditions. However, if the model excels in a particular clinical subgroup, it may suggest that patients in that subgroup are more suitable for our predictive signature. For instance, our prognostic signature showed superior predictive performance in patients with advanced HCC (stages II-IV) compared to those in the early stage (*p*<0.001 vs. *p*=0.015) (**Figures 4K, L**), indicating its suitability for advanced HCC patients. In summary, validation of our novel signature in clinical subgroups is a pivotal step, ensuring the reliability of research outcomes and providing profound insights for the practical application of the model in clinical practice.

### 3.4 The predictive value evaluation of the 5-DRLs signature, and the construction and validation of the predictive nomogram

**Figure 5:**
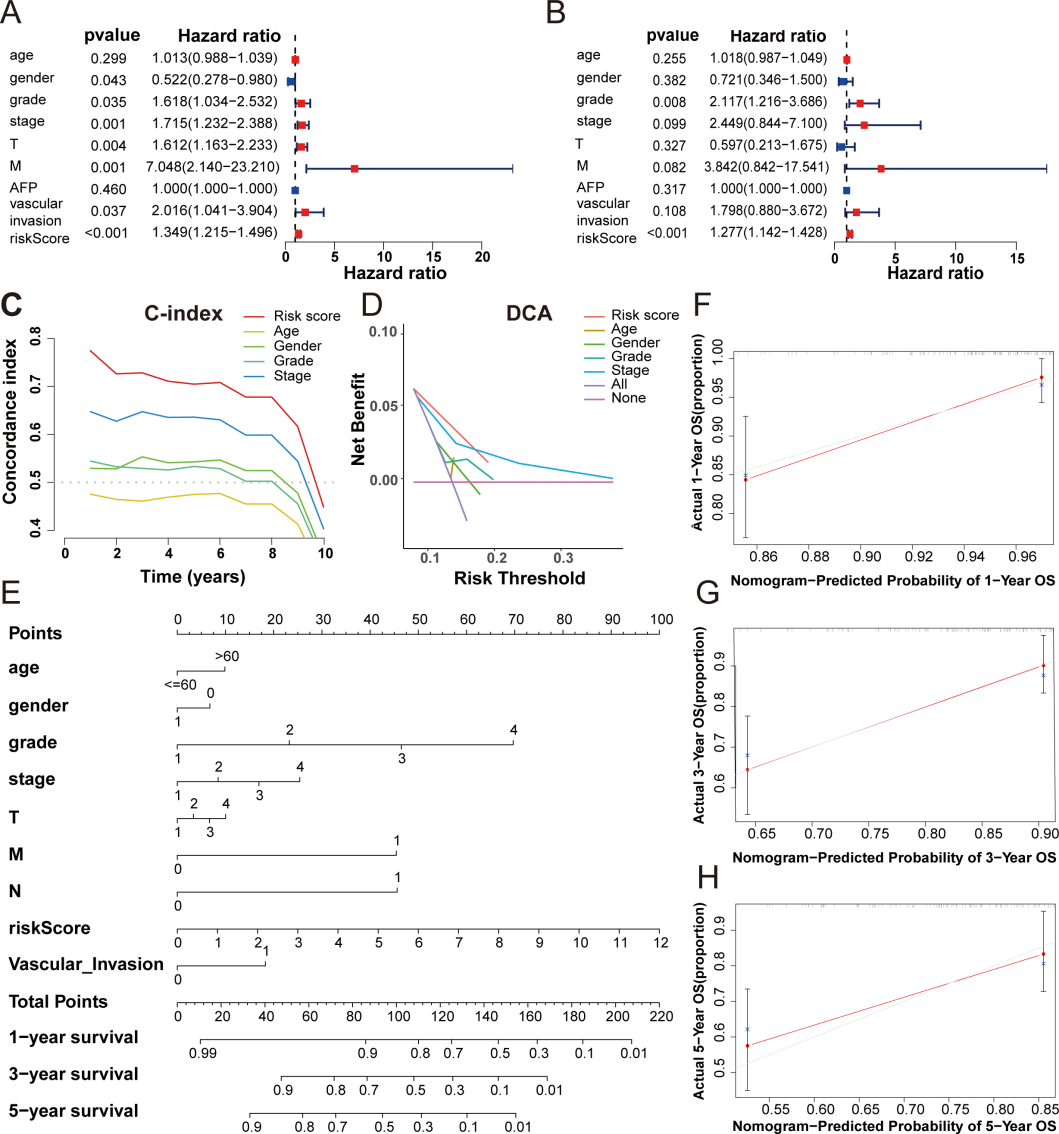
The predictive value evaluation of the 5-DRLs signature, and the construction and validation of the predictive nomogram. **(A, B)** The univariate cox and the multivariate cox regression analysis between risk score and other clinicopathological variables in HCC patients. **(C)** The concordance index of five indicators for OS in patients with HCC. **(D)** Decision‐curve analysis for five indicators for OS in patients with HCC. **(E)** A nomogram combined risk score with other clinicopathologic variables (age, gender, grade, pathological tumor stage, T stage, M stage and N stage and vascular invasion) to predict overall survival time in HCC patients. **(F-H)** Nomogram-predicted probability of 1, 3, 5-year OS.

Univariate and multivariate Cox regression analyses were used to explore whether the risk score calculated by the predictive signature could be an independent prognostic indicator for predicting the outcomes of HCC patients. Univariate Cox regression analysis showed that the risk score (hazard ratio [HR] = 1.349, 95%CI = 1.215-1.496, *p* < 0.001) was a prominent predictor of patients’ prognosis. In addition, gender, grade, stage, T stage, and M stage were all related to prognosis by univariate Cox regression analysis (**Figure 5A**). However, in the multivariate Cox regression analysis, only grade (HR=2.117, 95%CI=1.216-3.686, *p*< 0.008) and risk score (HR=1.277, 95%CI=1.142-1.148, *p*< 0.001) were significant predictors of patients’ prognosis (**Figure 5B**). The C-index was used to evaluate the discrimination ability of our predictive model. The risk score calculated by our signature exhibited a higher C-index than other clinical variables, underscoring the superiority of our signature (**Figure 5C**). Additionally, decision curve analysis (DCA) was employed to validate the performance of the prognostic signature. The positive clinical net benefit interval of the risk scores surpassed others in the risk threshold range of 0.1-0.2, which also indicated the superior performance of our five DRLs signature (**Figure 5D**).

A nomogram, a common tool to estimate the personal prognosis of tumors, was able to create an individual numerical probability of a clinical event by calculating many prognostic and crucial factors (31). To simplify our model into an easy numerical estimate of the probability of 1-, 3-, and 5-year OS of HCC, a nomogram based on age, gender, grade, stage, T stage, M stage, N stage, vascular invasion, and risk score was established (**Figure 5E**) (25). The 1-, 3-, and 5-year calibration curves revealed that the predictive outcome was close to the actual OS rate, suggesting a notable predictive value of our signature (**Figures 5F-H**).

### 3.5 5-DRLs prognostic signature for immune microenvironment and immunotherapy response discrimination in HCC

**Figure 6:**
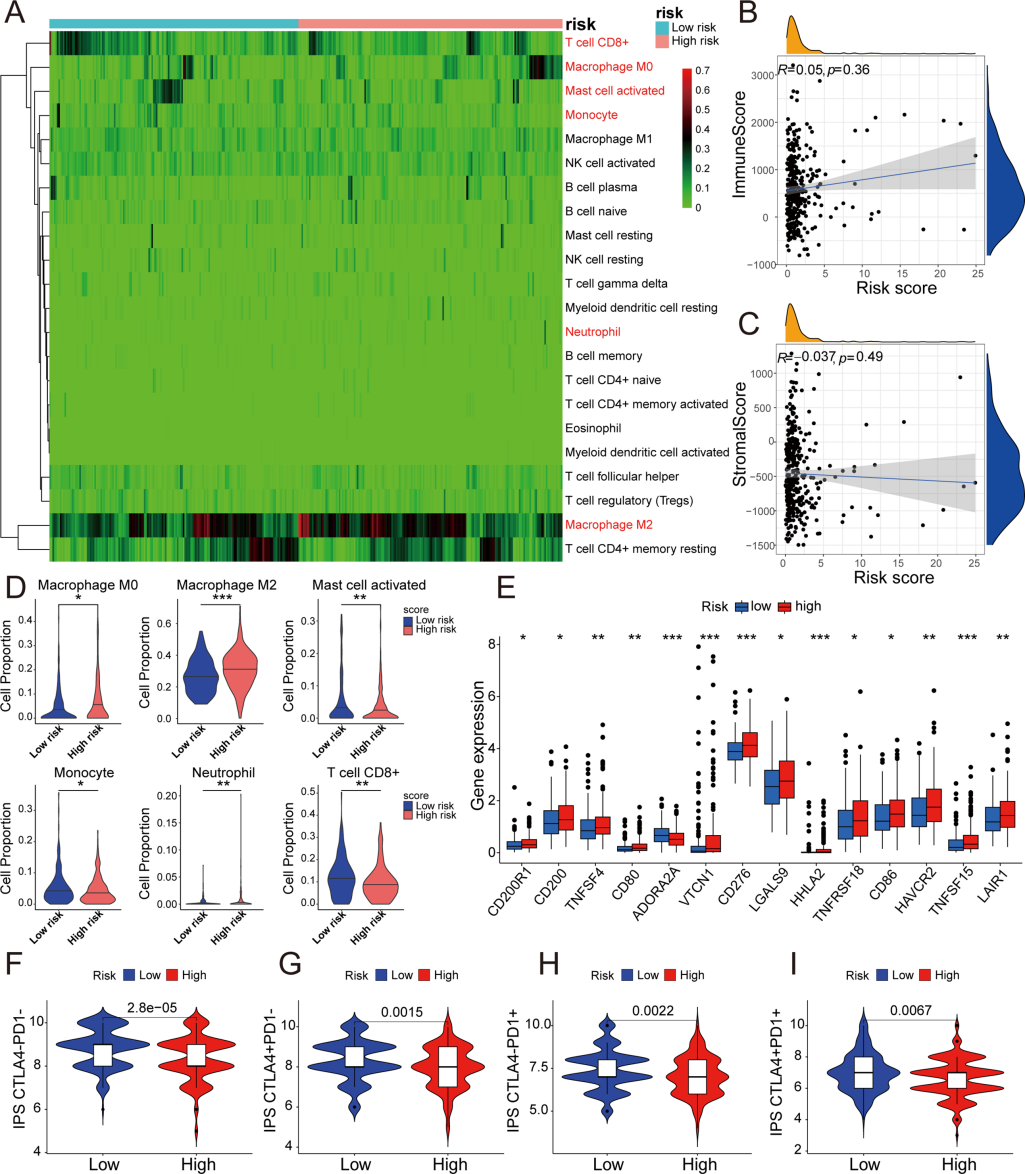
5-DRLs prognostic signature for immune microenvironment and immunotherapy response discrimination in HCC. **(A)** Relative proportion of 22 different immune cells based on CIBERSORT in the low-risk group and the high-risk group. Immune cells in red indicated that there was a significant difference between two groups. **(B)** The relationship between the risk score and immune Score. **(C)** The relationship between the risk score and Stromal Score. **(D)** The proportion of M0 macrophages, M2 macrophages, activated mast cell, monocyte, neutrophil, and CD8+ T cell in the low-risk group and the high-risk group. **(E)** Differential expressions of immune checkpoint genes between high- and low-risk groups. **(F-I)** Immunophenoscore predicts response to immunotherapy with CTLA-4 and PD-1 blockers. *p < 0.05, **p < 0.01, and ***p < 0.001.

Besides the crucial roles of gene mutations and epigenetic alterations in cancer, further investigations have found that the tumor immune microenvironment (TME) play an increasingly pivotal role in tumor physiology (32). The different characteristics of tumors were determined by distinctive stromal cell types and various sub-cell types (33). To explore the correlation between the 5-DRLs signature and TME, the CIBERSORT algorithm, which can estimate the abundance of immune cell types, was used (34). It could be found in the heatmap that various immune cells were significantly distinguished between the low-risk group and the high-risk group (**Figure 6A**). Specifically, M0 macrophages (*p* < 0.05), M2 macrophages (*p* < 0.001), and neutrophils (*p* < 0.01) were more abundant in the high-risk group, whereas CD8+ T cells (*p* < 0.01), activated mast cells (*p* < 0.01), and monocytes (*p* < 0.05) were more percentage in the low-risk group (**Figure 6D**). Spearman’s correlation test was used to determine the relationship between the immune score and risk score (R=0.05, *p*=0.36) and between the stromal score and risk score (R=0.037, *p*=0.49). However, neither had an apparent relationship (**Figures 6B, C**).

The expression levels of CD200R1, CD200, TNFSF4, CD80, VTCN1, CD276, LGALS9, HHLA2, TNFRSF18, CD86, HAVCR2, TNFSF15, and LAIR1 were all significantly higher in the high-risk group, with the exception of ADORA2A (**Figure 6E**). Remarkably, the expression of CD276 was considerably higher in the high-risk group than in the low-risk group (*p* < 0.001). Simultaneously, immunophenoscore (IPS), a score based on immunogenicity to predict immunotherapy potential, was used to assess the potential effects of two common immune treatment targets, CTLA-4 and PD-1 (35). As shown in the violin plot, IPS, IPS-CTLA4, IPS-PD1, and IPS-PD1+CTLA4+ were all significantly higher in the low-risk group than in the high-risk group. (*P*< 0.01) (**Figures 6F-I**). Therefore, the 5-DRLs signature established in this study has potential immunotherapy predictive value for clinical HCC treatment.

### 3.6 Correlation between 5-DRLs signature and TMB, and predictive analysis of drug sensitivity

**Figure 7:**
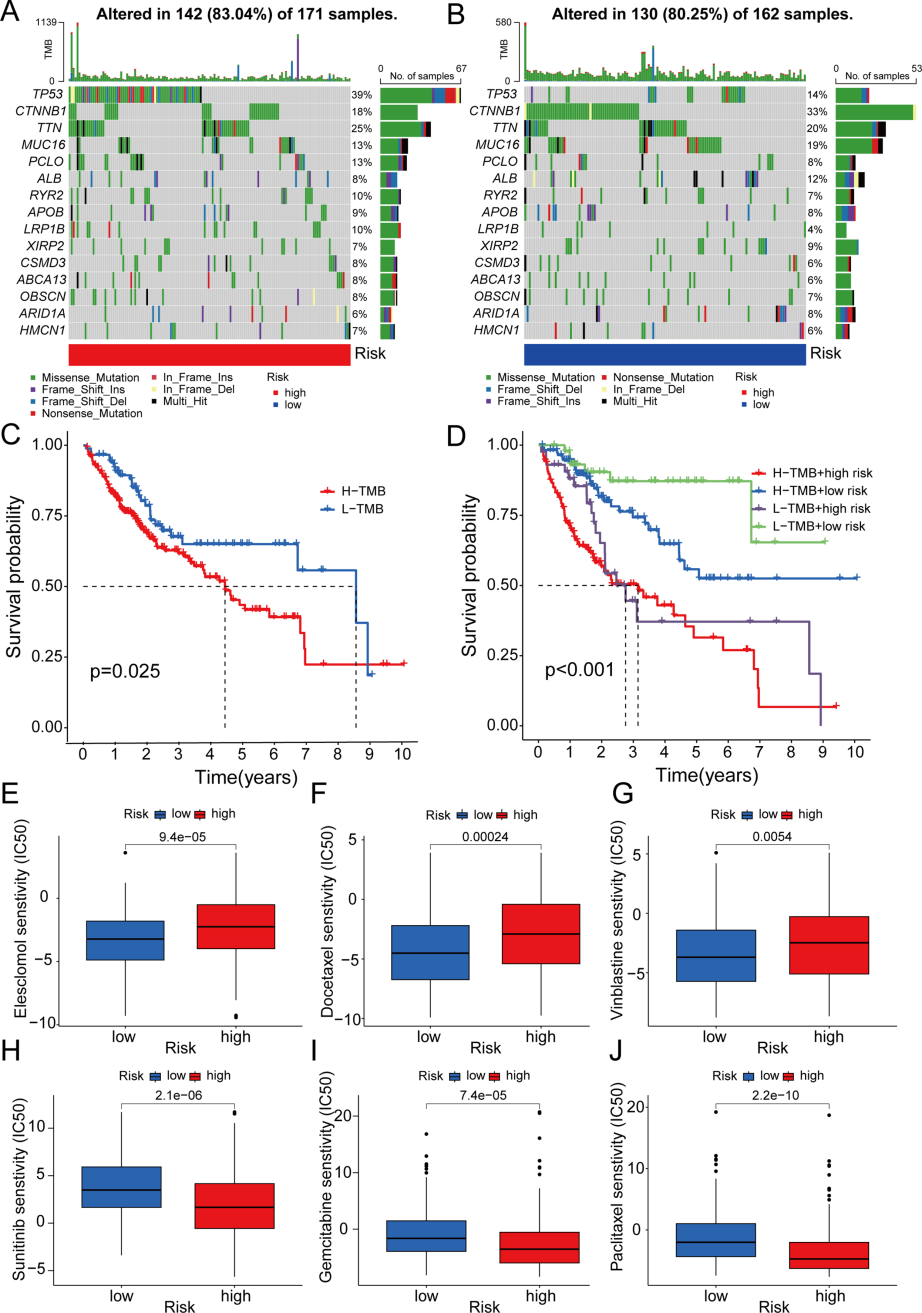
Correlation between 5-DRLs signature and TMB and drug sensitivity analysis. **(A, B)** Waterfall plot of the TMB in the high- and low-groups revealed the top 15 most frequent mutation genes. **(C)** The Kaplan–Meier curve for survival status and survival time in the high-and low-TMB groups. **(D)** The Kaplan–Meier curve for survival status and survival time in the high-TMB + high-risk, low-TMB + high-risk, high-TMB + low-risk and low-TMB + low-risk groups. **(E-J)** IC50 of elesclomol, docetaxel, vinblastine, sunitinib, gemcitabine and, paclitaxel between the two risk groups. TMB, tumor mutational burden; IC50, half-maximal inhibitory concentration.

Tumor mutation burden (TMB), which is based on the generation of immunogenic neoantigens from tumor gene mutations, had been regarded as a predictive biomarker for the response to immune checkpoint blockade (ICB) (36). Therefore, we analyzed the correlation between the 5-DRLs signature and TMB and found that their mutative frequencies were similar (high-risk group, 83.04%; low-risk group, 80.25%). Specifically, TP53 (39%), TTN (25%), CTNNB1(18%), MUC16(13%), and PCLO (13%) were the five most frequently mutated genes in the high-risk group, whereas CTNNB1(33%), TTN (20%), MUC16 (19%), TP53 (14%), and ALB (12%) were the top five genes in the low-risk group (**Figures 7A, B**). Meanwhile, we explored the OS rates between the high-TMB and low-TMB groups and further explored them by considering different risk scores separately. As depicted in **Figures 7C, D**, the high-TMB group had a relatively more unfavorable outcome (P<0.025) (**Figure 7C**). In addition, the low-TMB plus low-risk group was the most favorable one among the four groups (P<0.001) (**Figure 7D**).

To predict the potential for medical treatment and achieve precise individualized oncology therapy, drug-sensitivity analysis based on the different risk groups was performed. The IC50 values of Elesclomol, Docetaxel and Vinblastine were lower in the low-risk group than in the high-risk group, which indicated that they were probably more sensitive to low-risk HCC patients (**Figures 7E-G**). Conversely, the IC50 values for Sunitinib, Gemcitabine and Paclitaxel were higher in the low-risk group, suggesting a higher likelihood of obtaining better responses in the high-risk group (**Figures 7H-J**).

### 3.7 Pathway and functional enrichment analyses of DEGs

**Figure 8:**
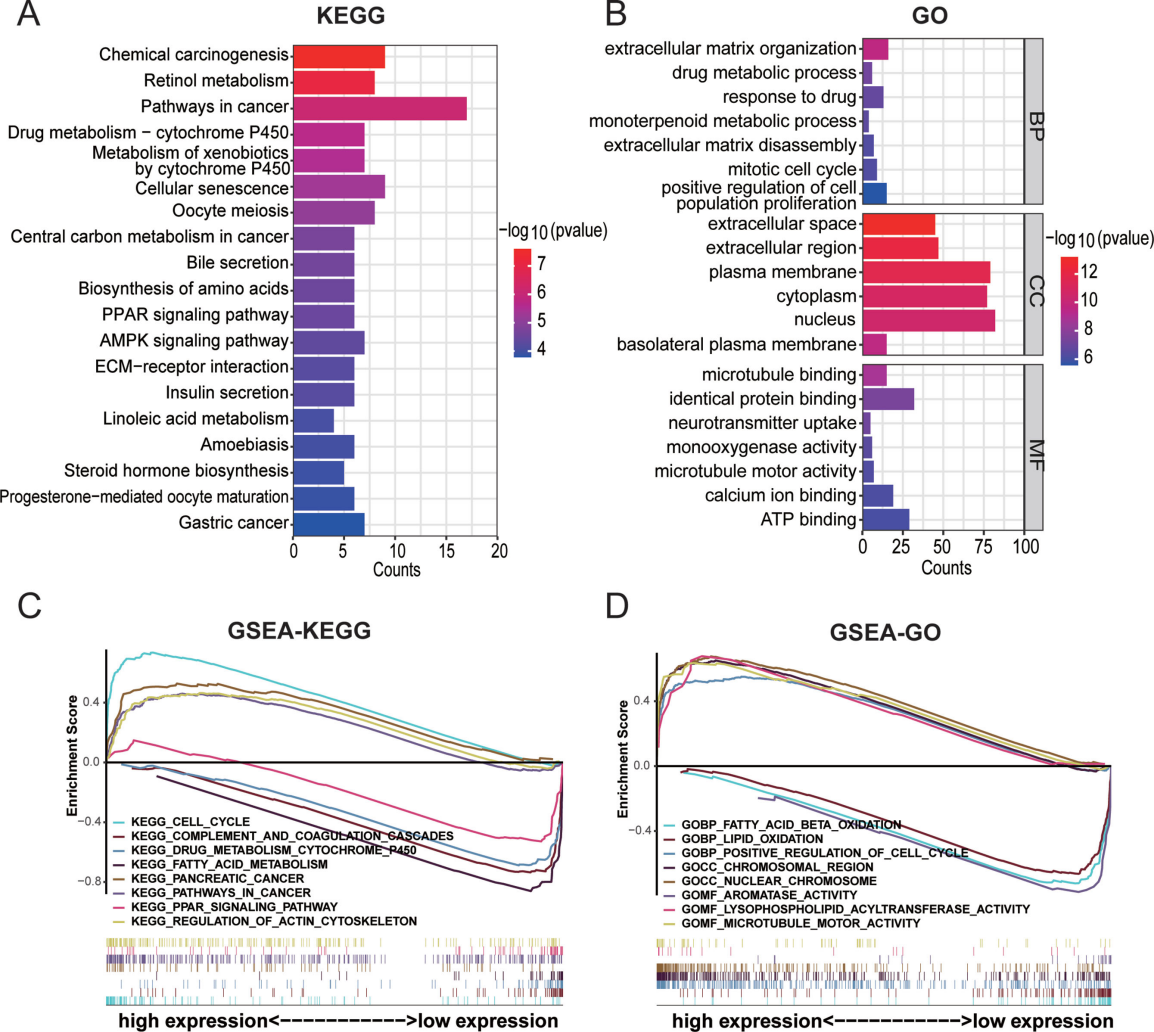
Pathway and functional enrichment analyses of DEGs between high- and low- risk group. **(A)** KEGG pathway enrichment of DEGs between high- and low- risk group. **(B)** GO functional annotation analysis of DEGs between high- and low- risk group. **(C, D)** Gene set enrichment analysis between high- and low- risk group.

To elucidate the potentially different mechanisms in the high- and low-risk groups, we selected differentially expressed genes (DEGs). KEGG pathway enrichment and GO functional annotation analyses were conducted based on the DEGs between the low- and high-risk groups. In KEGG pathway enrichment, many signaling pathways were significantly enriched, and the top 20 involved pathways, including pathways in cancer, cellular senescence, PPAR signaling pathway, and ECM-receptor interaction pathway, were depicted (**Figure 8A**). The top 100 pathways in the KEGG analysis of the DEGs were shown in **Supplementary Table 5**. The top 20 pathways enriched by GO functional annotation analysis of DEGs were shown in **Figure 8B**, which show that pathways such as extracellular matrix organization, nucleus, and identical protein binding were enriched. The top 100 pathways in the GO analysis of the DEGs were shown in **Supplementary Table 6**.

Furthermore, GSEA was performed to reveal potential biological processes and mechanistic pathways between the different risk groups. The top 100 pathways in the GSEA-KEGG and GSEA-GO analyses between the high- and low-risk groups were shown in **Supplementary Table 7**. Surprisingly, it was revealed that the regulation of the actin cytoskelet.

on and microtubule motor activity were enriched in the high-risk group (**Figures 8C, D**). Previous studies had demonstrated that actin, microtubules, and intermediate filaments were integral components of the cytoskeleton. The formation of disulfide bonds in actin cytoskeleton proteins that led to F-actin collapse and generation of forces within cells, ultimately inducing disulfidptosis (17, 37). Hence, it could be inferred that the 5-DRLs signature is closely associated with disulfidptosis.

### 3.8 Verification of 5 DRLs expressions in HCC cell lines and tissues

**Figure 9:**
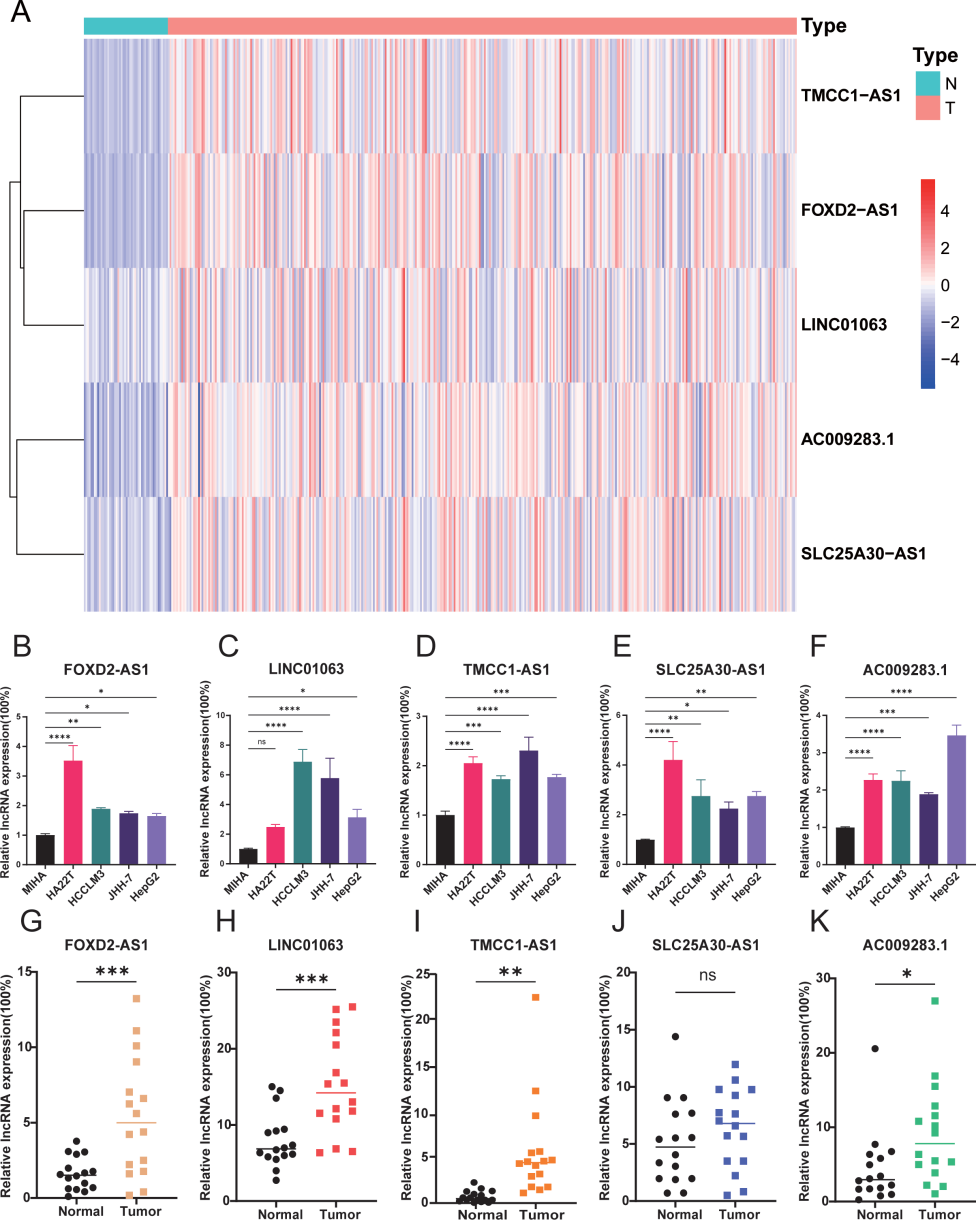
Verification of 5 DRLs expressions in HCC cell lines and tissues. **(A)** The heatmap of the expression of 5 DRLs in HCC tissues and adjacent tissues from TCGA database. **(B-F)** 5 lncRNAs’ (FOXD2-AS1, LINC01063, TMCC1-AS1, SLC25A30-AS1, AC009283.1) expression levels in liver cell lines and different HCC cell lines including MIHA, HA22T, HCCLM3, JHH-7, HepG2 (n=3) (One-way ANOVA). **(G-K)** 5 lncRNAs’ expression levels in HCC tissues and adjacent normal tissues (T-test). *p < 0.05, **p < 0.01, ***p < 0.001 and ****p<0.0001. ns, no significance.

According to the data presented in **Figure 9A**, there were significantly higher expression levels of the five DRLs in HCC tissues than in normal tissues from TCGA database. To further verify this finding, we conducted experiments using a normal liver cell line MIHA and four distinct liver cancer cell lines: HA22T, JHH-7, HCCLM3, and HepG2. Subsequently, we extracted RNA from each cell line and used RT-qPCR to confirm the expression levels of these lncRNAs. The results demonstrated that the five DRLs exhibited higher expression levels in HCC cells than in normal liver cells (**Figures 9B-F**). For further corroboration, we collected 16 pairs of HCC tissues and adjacent normal tissues from patients with HCC who had undergone surgical resection. Similar results were observed in these clinical samples, indicating higher expression levels of the aforementioned lncRNAs in HCC tissues, except SLC25A30-AS1 (**Figures 9G-K**).

### 3.9 Validation of disulfidptosis phenotype in HCC cell lines

**Figure 10:**
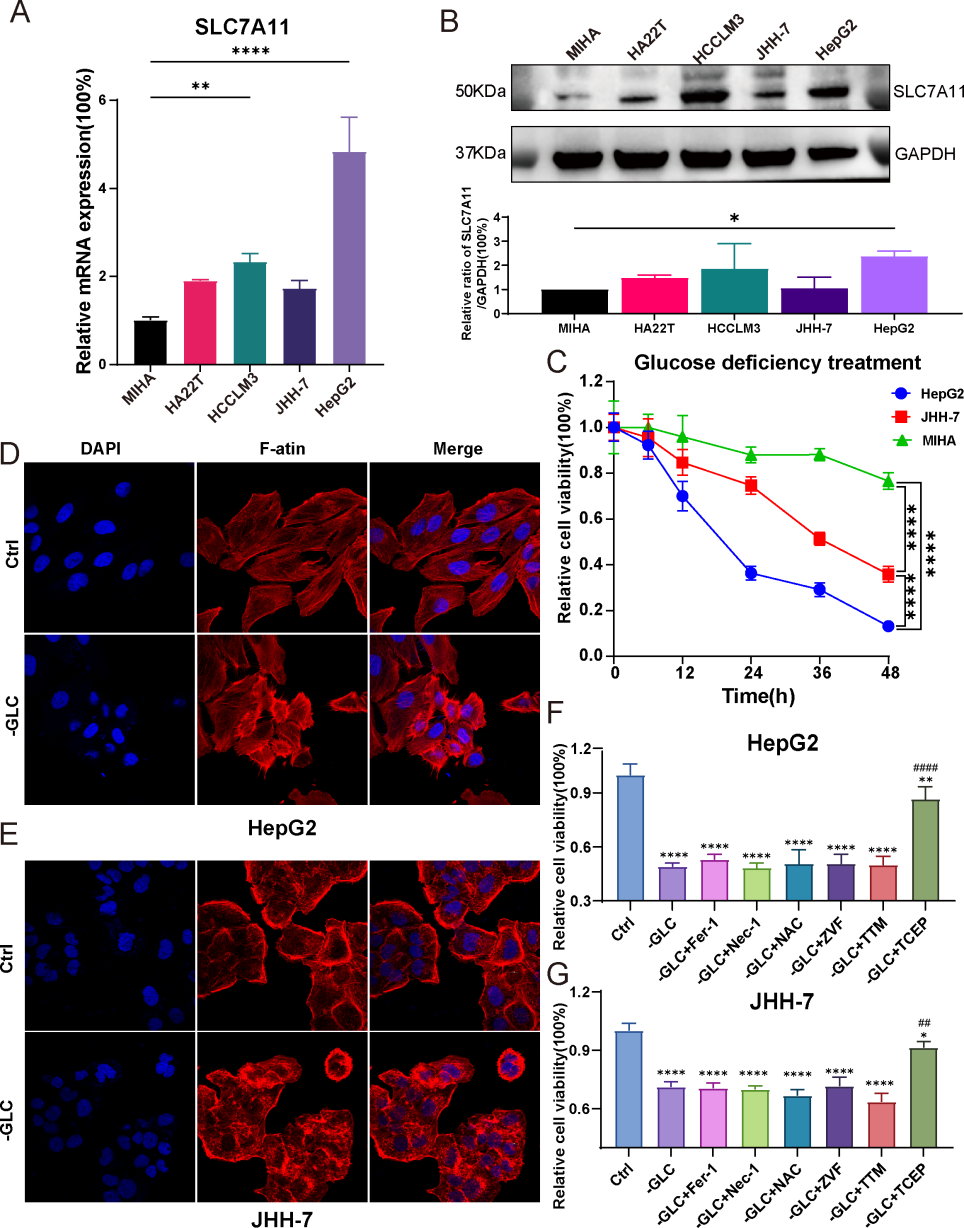
Validation of disulfidptosis phenotype between HCC cell lines. **(A)** The expression levels of SLC7A11 gene in 5 cell lines (n=3) (One-way ANOVA). **(B)**The Western blot bands of SLC7A11 and GAPDH protein in 5 cell lines (n=3) (One-way ANOVA). **(C)** Cell viability curve of HepG2 and JHH-7 about 6, 12, 18 and 24 h after glucose starvation treatment, respectively (Two-way ANOVA). **(D, E)** F-actin staining by phalloidin of HepG2 and JHH-7. **(F, G)** The rescue effect of cell death inhibitors in HepG2 and JHH-7 undergoing disulfidptosis for 12 hours was explored through CCK8 assay (One-way ANOVA). ZVF, Z-VAD-FMK; Fer-1, ferrostatin-1; Nec-1, necrostatin-1; NAC, N-acetyl cysteine; TTM, Tetra thiomolybdate; TCEP, Tris(2-carboxyethyl) phosphine. * *p* < 0.05, **p < 0.01, and ****p < 0.0001. ##p<0.01, ####p<0.0001. (*means compared with group Ctrl; #means compared with group -GLC).

To verify the phenomenon of disulfidptosis could be repeated in HCC, we induced disulfidoptosis in selected HCC cell lines through glucose deprivation. Initially, we compared the expression levels of SLC7A11 gene among different liver cell lines and HCC cell lines in mRNA level and protein level. The result revealed that all of HCC cell lines exhibited higher expression levels than the normal liver cell line MIHA. Among the HCC cell lines, HepG2 and HCCLM3 displayed significantly elevated expression, whereas HA22T and JHH-7 had relatively lower levels (**Figures 10A, B**). Subsequently, we selected HepG2, characterized by high SLC7A11 expression level, and JHH-7, characterized by low SLC7A11 expression level, to validate the disulfidoptosis phenotype in HCC. The cell viability curve indicated that HepG2 was more sensitive to disulfidoptosis (**Figure 10C**). Besides, after exposing both cell lines in glucose deprivation for 12 hours, we fixed those cells in 4% formaldehyde and stained them with phalloidin. HepG2 cells exhibited substantially morphological changes, including intensified cytoskeletal staining. Compared with normal cells, treated cells displayed contraction, actin filament accumulation and reduced cell volume (**Figure 10D**). Relatively lesser changes were observed in JHH-7 cells (**Figure 10E**).

We also explored the expression of ten disulfidptosis-related genes in the TCGA database and in 16 HCC patients. As shown in **Supplementary Figures 5** and **6**, we presented the expression of these ten genes in TCGA and HCC patients, respectively. It was observed that most of the disulfidptosis-related genes were more highly expressed in HCC tissues compared to normal tissues. This indicates that these genes could potentially be targeted to induce disulfidptosis and serve as treatment targets for HCC in future.

To further confirm that cell death induced by glucose deficiency was not caused by other forms of cell death, we introduced various cell death inhibitors, such as Ferrostatin-1 (Fer-1, a ferroptosis inhibitor), Necrostatin-1 (Nec-1, a necroptosis inhibitor), Z-VAD-FMK (ZVF, an apoptosis inhibitor), N-acetyl cysteine (NAC, an antioxidant), Tetra thiomolybdate (TTM, a cuproptosis inhibitor) to the treatment groups during the 12-hour glucose starvation and detected the cell viability. However, these inhibitors did not alleviate cells death. Surprisingly, the addition of Tris(2-carboxyethyl) phosphine (TCEP), a non-thiol reducing agent, resulted in a remarkable rescue in cell death (**Figures 10F, G**). In summary, our study conclusively showed that glucose deprivation could trigger disulfidptosis in HCC. The extent of this novel cell death was directly correlated with the expression level of the SLC7A11 gene. Besides, this cellular damage resulting from sulfide accumulation could be mitigated by TCEP.

### 3.10 Disulfidptosis regulated by LINC01063 and FOXD2-AS1

According to the disulfidptosis-related lncRNA signature, five lncRNAs (AC009283.1, SLC25A30-AS1, FOXD2-AS1, LINC01063, and TMCC1-AS1) were identified as central components within this network, suggesting their potential significance in the disulfidptosis of HCC.

**Figure 11:**
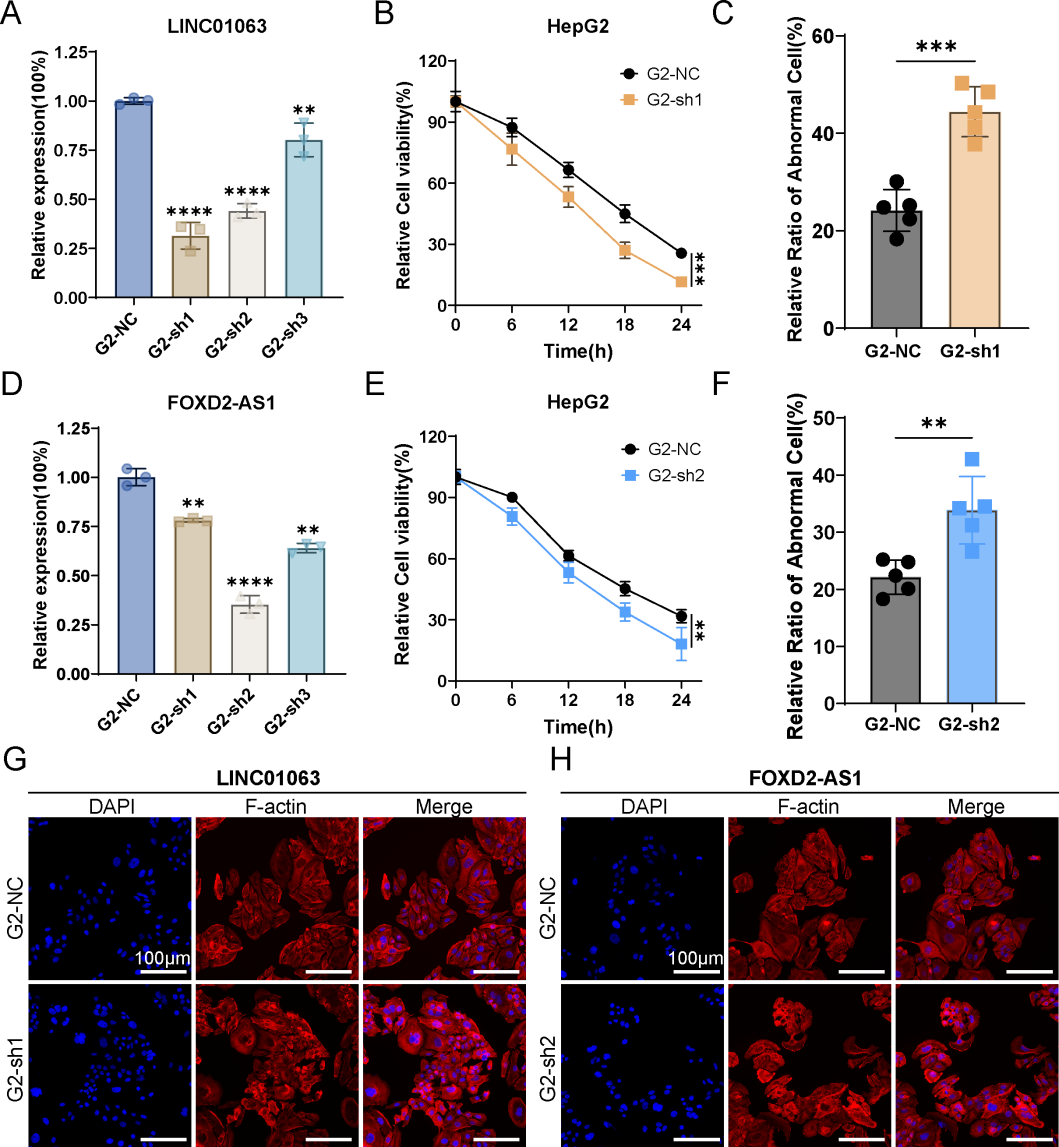
LINC01063 and FOXD2-AS knockdown increases susceptibility to disulfidptosis in HepG2 cells. **(A, D)** The expression levels of LINC01063 and FOXD2-AS lncRNAs in HepG2 cells, with or without siRNA treatment, are shown (n=3) (One-way ANOVA). **(B, E)** HepG2 cells with or without LINC01063 and FOXD2-AS knockdown were subjected to glucose starvation for 0, 6, 12, 18, and 24 hours, followed by assessment using the CCK8 reagent (Two-way ANOVA). **(C, F)** The proportion of abnormal cells in different groups was quantified using F-actin staining images obtained with a fluorescence microscope (T-test). **(G, H)** Representative fluorescence images of F-actin staining after 12 hours of glucose starvation are shown for HepG2 cells with or without LINC01063 and FOXD2-AS knockdown. **p < 0.01, ***p < 0.001 and ****p < 0.0001.

We selected two lncRNAs, FOXD2-AS1 and LINC01063, for further investigation. Initially, we designed silencing RNAs to knock down the expression of FOXD2-AS1 and LINC01063 in the HepG2 cell line. The knockdown efficiency was confirmed via RT-qPCR, revealing that LIN010613 siRNA1 and FOXD2-AS1 siRNA2 achieved greater than 70% knockdown efficiency (**Figures 11A, D**). Subsequent cell viability assays demonstrated that the depletion of FOXD2-AS1 and LINC01063 sensitized HCC cells to disulfidptosis (**Figures 11B, E**). After 24 hours of glucose starvation, cells with LINC01063 and FOXD2-AS1 knockdown showed significantly lower viability compared to the control group. Additionally, fluorescence microscopy with F-actin staining further demonstrated that, after 12 hours of glucose starvation, cells with LINC01063 and FOXD2-AS1 knockdowns displayed more markedly abnormal cell morphology (**Figures 11C, F**). Specifically, these HepG2 cells exhibited substantial morphological changes, including intensified cytoskeletal staining, pronounced cytoskeletal shrinkage, actin filament accumulation, and reduced cell volume (**Figures 11G, H**).

These findings corroborate our bioinformatics analysis, which indicated that the lncRNAs within the prognostic signature play a regulatory role in disulfidptosis. Specifically, the downregulation of lncRNAs such as LINC01063 and FOXD2-AS1 enhances the sensitivity of HCC cells to disulfidptosis. The precise regulatory mechanisms underlying this effect warrant further investigation.

## 4 Discussion

HCC is the sixth most common cancer worldwide and is known for its high mortality rate and aggressiveness (38). Therefore, understanding the pathogenesis of HCC and exploring new diagnostic and prognostic markers are crucial. Meanwhile, disulfidptosis has provided a new theoretical foundation for the development of innovative antitumor treatments (17). In this study, we conducted a comprehensive analysis of the transcriptional expression of 10 disulfidptosis-related genes (DRGs) in HCC patients based on the TCGA database. Subsequently, we identified the co-expressed lncRNAs associated with these DRGs and developed a novel scoring system based on five co-expressed prognostic-related lncRNAs (FOXD2-AS1, SLC25A30-AS1, TMCC1-AS1, LINC01063, and AC009283.1). ROC, C-index, and DCA analyses revealed that the risk signature had high accuracy and excellent sensitivity. Moreover, univariate and multivariate Cox analyses confirmed it to be an independent prognostic factor for patients according to univariate and multivariate Cox analyses. In addition, we created a nomogram by combining the risk score with other clinicopathological features. This nomogram provided an intuitive and quick individualized risk assessment for patients with HCC. We established a signature of lncRNAs associated with disulfidptosis, providing a potential strategy for guiding individualized treatment and contributing to the prediction of prognosis and immune response in HCC patients. Finally, we validated the relative expression of the five lncRNAs in both cell lines and HCC tissues and verified the disulfidptosis phenotype of HCC under glucose deprivation.

The five DRLs, components of our signature, were identified as potentially associated with HCC and disulfidptosis in the existing literature. We discovered that these lncRNAs had been previously studied in various types of cancers. For instance, Miranda’s research indicated that lncRNA AC009283.1 may be causally related to carcinogenesis. It has been suggested that AC009283.1, contributes to the malignant phenotype of the HER2-rich subtype of breast cancer, leading to an upregulation of tumor cell proliferation capacity and resistance to apoptosis (39). However, in our risk signature, AC009283.1 exhibited higher expression levels in the lower-risk group (**Figure 4A**). The heterogeneity of tumors may be responsible for this inconsistency. For LINC01063, Xu’s al. reported that it acted as an oncogene in melanoma by functioning as a sponge for miR-5194, leading to increased cancer cell proliferation, migration, invasion, and epithelial-mesenchymal transition (40). TMCC1-AS1 has also been implicated as a tumor promoter; its suppression led to increased E-cadherin expression and decreased proliferating cell nuclear antigen Ki67 expression in HCC cells (41). Mechanistic insights into FOXD2-AS1 have been extensively explored in gastric cancer, colorectal cancer, breast cancer, and other malignancies, primarily focusing on its cancer-promoting properties. For example, Xu et al. revealed that the knockdown of FOXD2-AS1 reduced transmembrane protein 9 (TMEM9) expression and increased the sensitivity of HCC cells to sorafenib (42). In addition, SLC25A30-AS1 showed a lower expression level in the high-risk group of our risk signature, which correlated with poor prognosis (**Figure 4A**). Hence, further experiments are required to elucidate how SLC25A30-AS1 regulates the malignant behavior of HCC.

TMB, associated with neoantigens present on the surface of cancerous cells, complements conventional biomarkers for predicting the effectiveness of ICB (43). Previous studies have reported that patients with high TMB tend to have poorer survival and better response to ICB (44). In this study, there was a significant disparity in TP53 and CTNNB1 gene mutations between the two groups: the high-risk group exhibited a higher frequency of TP53 gene mutations, whereas the low-risk group showed an elevated frequency of CTNNB1 gene mutations. CTNNB1-mutated HCC has been proven to be a homogeneous subtype of non-proliferative tumors with well-differentiated characteristics such as an intact tumor capsule, cholestasis, microtrabecular, and pseudoglandular architectural patterns (45, 46). Conversely, TP53-mutated tumors were poorly differentiated, with a compact pattern, multinucleated and pleomorphic cells, and vascular invasion (46). Additionally, previous studies have shown that TP53 could inhibit the expression of SLC7A11 (a key component of the cystine/glutamate antiporter), reducing the uptake of cystine and synthesis of cysteine-dependent glutathione (GSH), destroying cellular antioxidant defenses, ultimately accelerating ROS accumulation, and inducing ferroptosis (15, 47). However, high expression of SLC7A11 combined with glucose starvation could result in disulfidptosis (47, 48). Therefore, the cellular and molecular mechanism of how TP53 regulated the disulfidptosis and the balance between disulfidptosis and ferroptosis require further exploration.

Tumor cells under continuous evolution driven by constant selection and mutual interaction within the entire cellular ecosystem, ultimately giving rise to adaptive cellular phenotypes within the tumor microenvironment (TME) (49, 50). In our study, M2 macrophages were highly recruited to the high-risk group. Macrophages, which are versatile and heterogeneous innate immune cells, possessed plasticity that allows them to interact with a wide range of cell type including tumor cells, T lymphocytes, endothelial cells (ECs), and fibroblasts. This interaction can subsequently promote tumor tolerance and progression (51). Recent research has revealed a correlation between an unfavorable prognosis and M0 macrophages in HCC (52). In addition, there were two distinct types of polarized macrophages. Type 1-polarized macrophages (M1), identified by the expression of CD80, CD86, MHC II, iNOS, and CD68, were phagocytic and could impede tumor progression. In contrast, type 2-polarized macrophages (M2), induced under the influence of IL-4, IL-13, IL-10, and M-CSF, were immunosuppressive cells characterized by the expression of CD206, CD204, VEGF, CD163, and Arg-1. These actions can suppress the anti-cancer immune response (32, 53). Therefore, the high-risk group with higher recruitment of M2 macrophages in our study may have a relatively worse anticancer immune response, highlighting the predictive value of the prognostic signature in the TME.

In recent years, immune checkpoint inhibitors have been vigorously developed for cancer therapy. Atezolizumab plus Bevacizumab and Tremelimumab plus Durvalumab have been widely approved as standard-of-care first-line therapies for HCC (54). Tremelimumab, an anti-CTLA-4 antibody, inhibit the interaction between CTLA-4 and B7-1 (CD80) and CTLA-4 and B7-2 (CD86), reactivating T lymphocytes (55). As illustrated in **Figure 6E**, the gene expression of CD80 and CD86 was higher in the high-risk group, suggesting that Tremelimumab may be more effective in this group. In addition, the expression of CD276 (B7-H3) was significantly higher in the high-risk group. CD276, which is selectively expressed in tumor and immune cells, was associated with tumor cell proliferation, metastasis, and therapeutic resistance (56). Therefore, our 5-DRLs signature may have the potential to predict the expression of immune checkpoint genes and related immunotherapeutic responses.

However, this study remained several limitations. Firstly, our analysis relied on retrospective patient information available from public datasets. No external database was available to validate the reliability of the signature in terms of lncRNA expression and clinical prognostic data. Additional verification using prospective multicenter real-world data is required for this risk signature. Secondly, we only verified the differential expression of five lncRNAs in HCC compared to normal subjects using a small sample size. Thirdly, we only confirmed that disulfidptosis could be induced in HCC, without further investigation into the mechanisms and applications of this signature. Therefore, further studies are needed to thoroughly elucidate the function of disulfidptosis-related lncRNAs in HCC in future research.

## 5 Conclusion

In summary, we developed a novel 5-DRLs signature with excellent specificity and sensitivity, serving as a reliable prognostic indicator for patients with HCC. The nomogram, which includes age, clinical TNM staging, and risk scores, provides a straightforward tool for predicting the survival period of patients with HCC. Additionally, our signature has the potential to predict the effectiveness of immunotherapy and targeted therapies. We believe that our signature can build a bridge between HCC and disulfidptosis, ultimately serving as a clinically applicable diagnostic and therapeutic tool.

## Data Availability

The original contributions presented in the study are included in the article/**Supplementary Material**. Further inquiries can be directed to the corresponding authors.
