# Supplementary figures and images for: A disulfidptosis-related lncRNA signature for analyzing tumor microenvironment and clinical prognosis in hepatocellular carcinoma
Source: Front Immunol. 2024 Oct 7;15:1412277. doi: 10.3389/fimmu.2024.1412277 (PMC11491388; doi:10.3389/fimmu.2024.1412277)

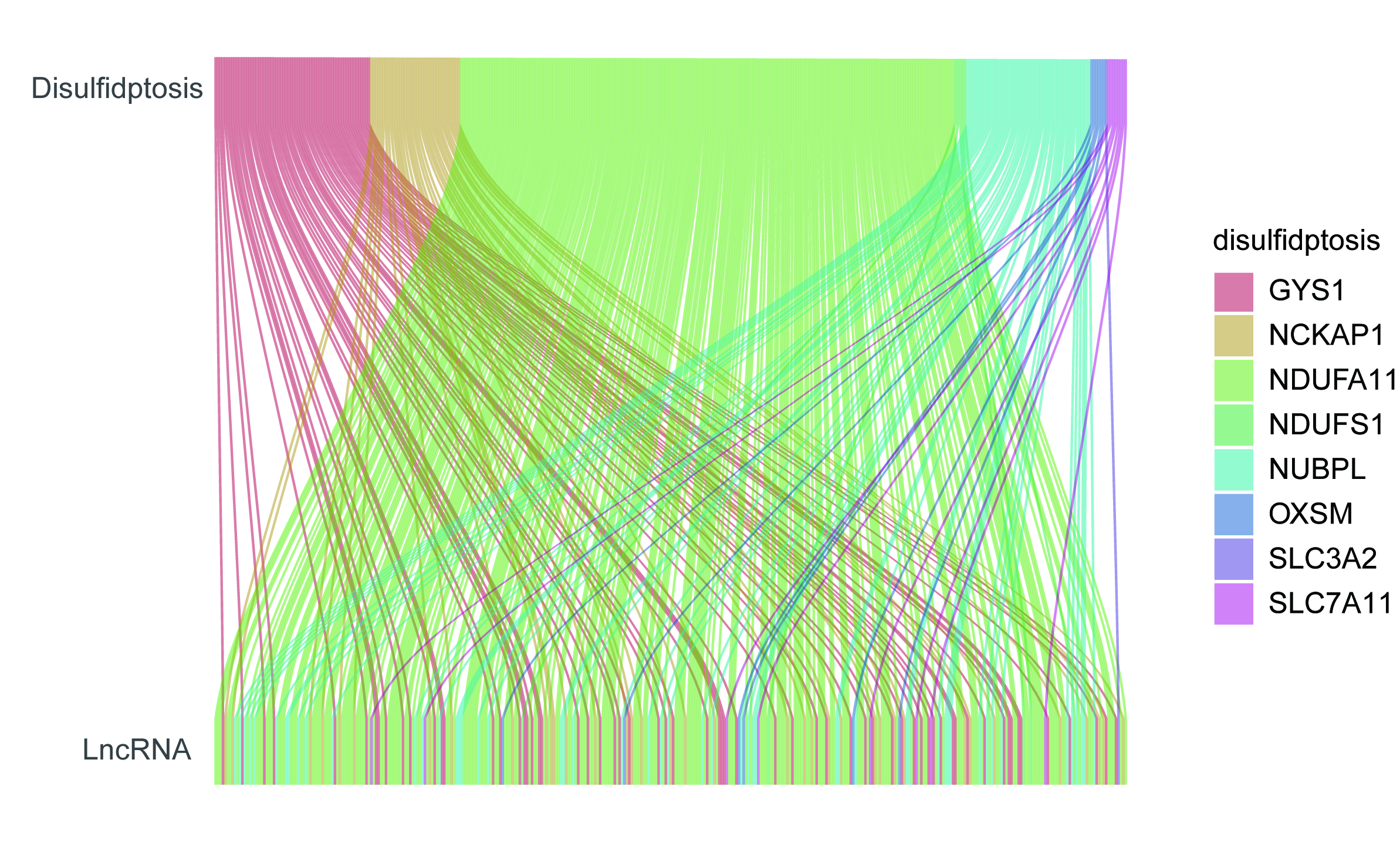

Supplement: Supplementary file 1 [file Image1.tif]

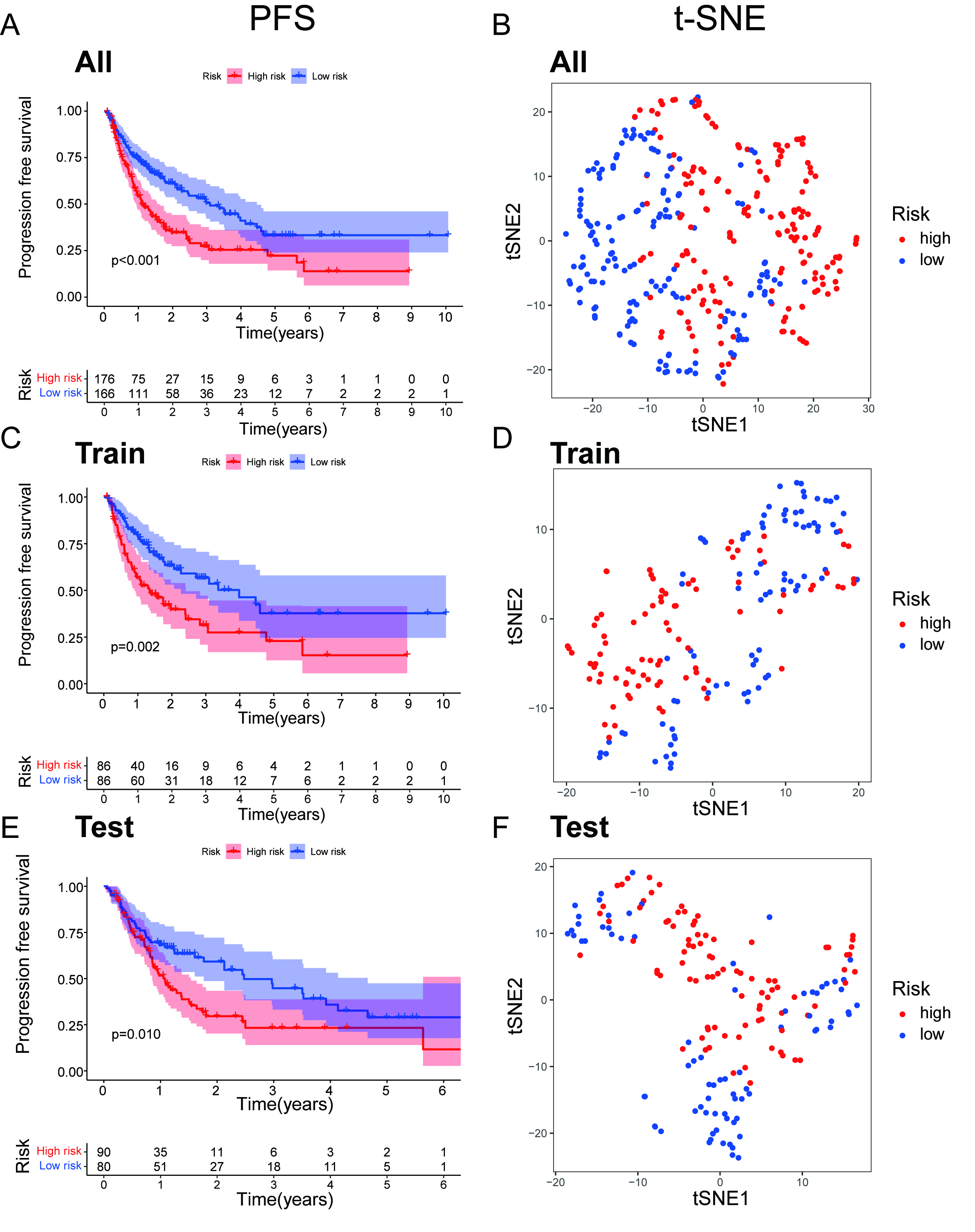

Supplement: Supplementary file 2 [file Image2.tif]

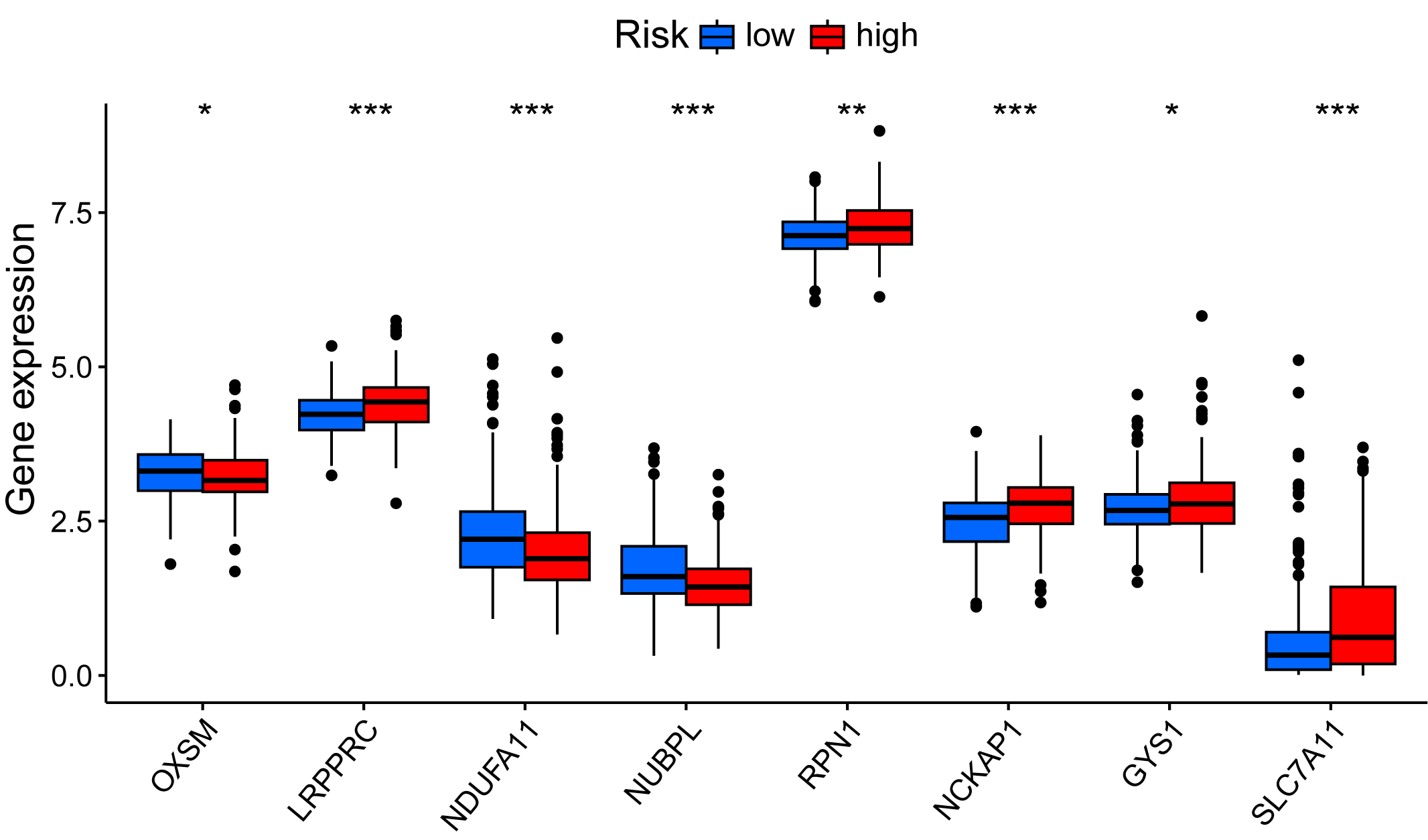

Supplement: Supplementary file 3 [file Image3.tif]

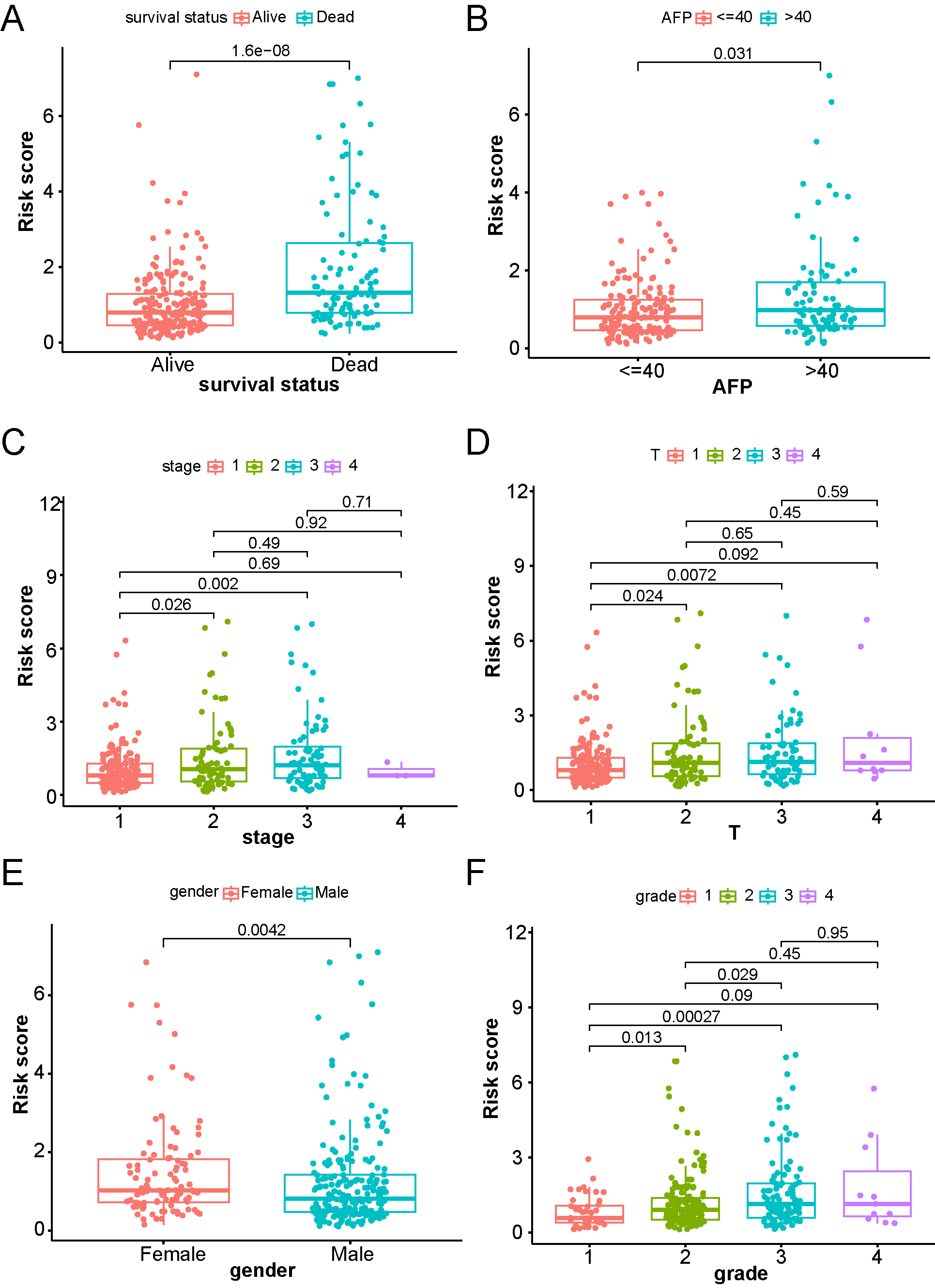

Supplement: Supplementary file 4 [file Image4.tif]

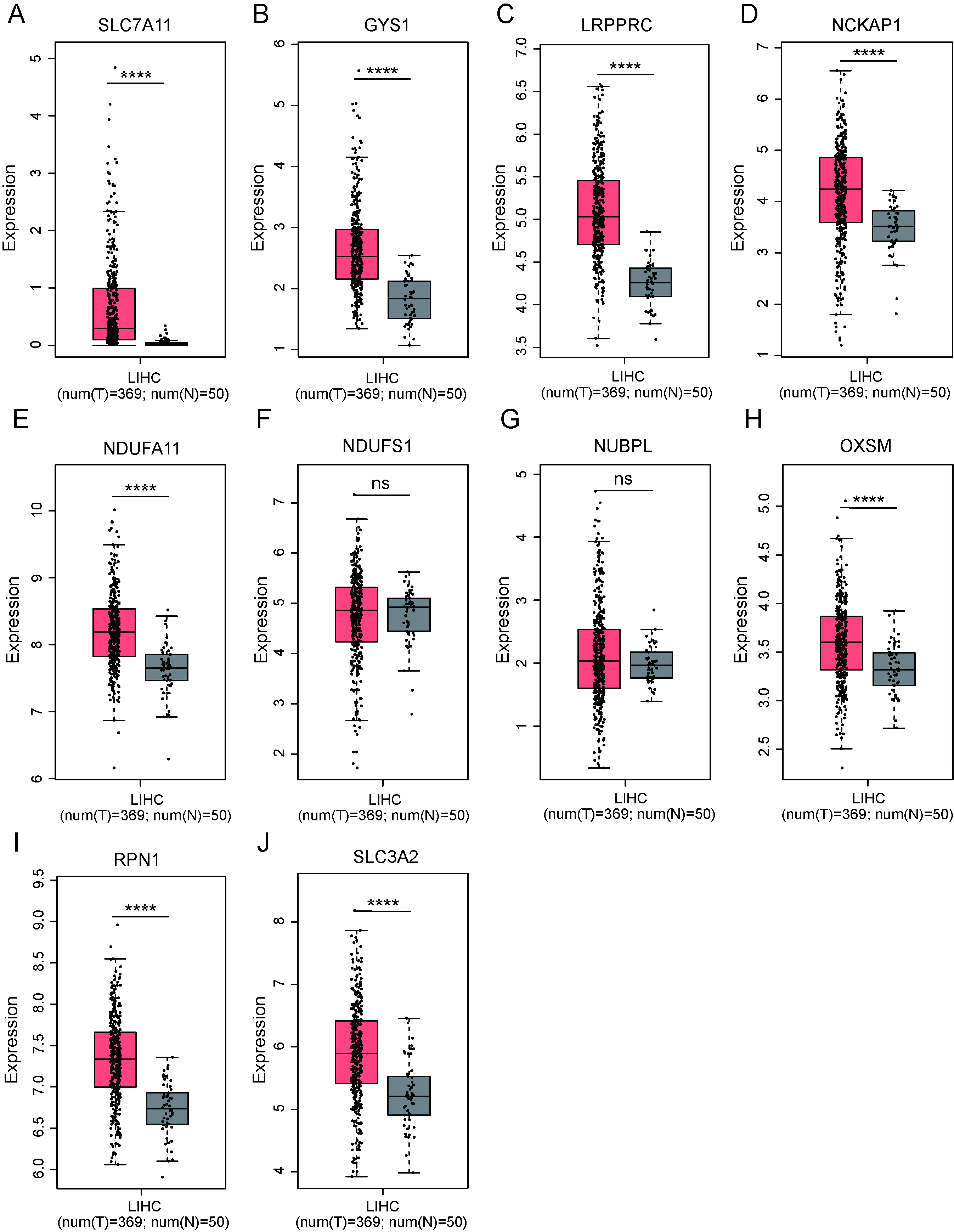

Supplement: Supplementary file 5 [file Image5.tif]

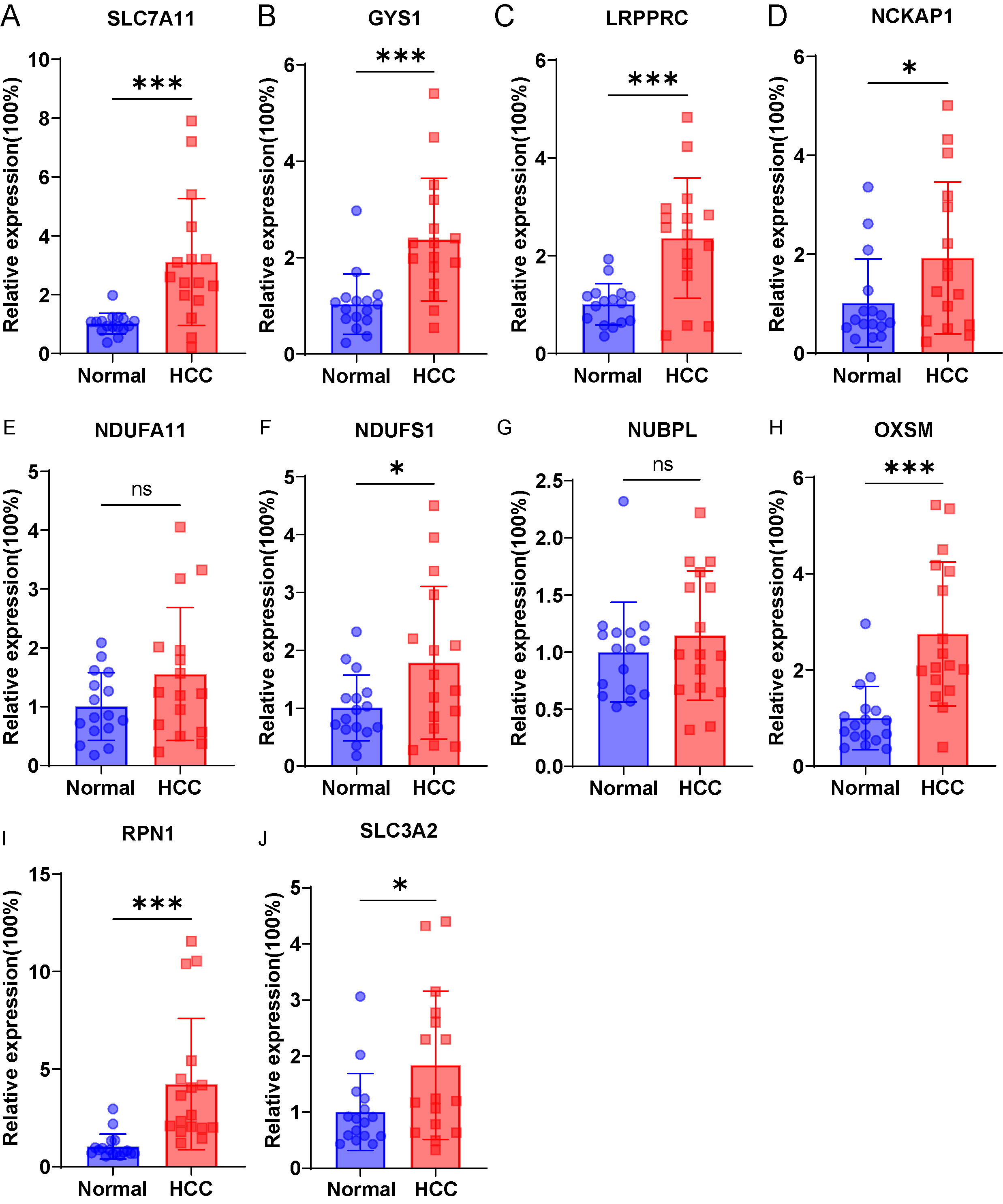

Supplement: Supplementary file 6 [file Image6.tif]
